# An update on vitamin B12-related gene polymorphisms and B12 status

**DOI:** 10.1186/s12263-018-0591-9

**Published:** 2018-02-06

**Authors:** S. Surendran, A. Adaikalakoteswari, P. Saravanan, I. A. Shatwaan, J. A. Lovegrove, K. S. Vimaleswaran

**Affiliations:** 1 Hugh Sinclair Unit of Human Nutrition, Department of Food and Nutritional Sciences and Institute for Cardiovascular and Metabolic Research (ICMR), University of Reading, PO Box 226, Whiteknights, Reading, RG6 6AP UK; 2 Warwick Medical School - Population Evidence and Technologies, University of Warwick, Coventry, CV4 7AL UK; 3 UK Academic Department of Diabetes and Metabolism, George Eliot Hospital, Nuneaton, UK

**Keywords:** Vitamin B12, Vitamin B12 levels, Cobalamin, Genetic epidemiology, Polymorphisms, Genetics of vitamin B12

## Abstract

**Background:**

Vitamin B12 is an essential micronutrient in humans needed for health maintenance. Deficiency of vitamin B12 has been linked to dietary, environmental and genetic factors. Evidence for the genetic basis of vitamin B12 status is poorly understood. However, advancements in genomic techniques have increased the knowledge-base of the genetics of vitamin B12 status. Based on the candidate gene and genome-wide association (GWA) studies, associations between genetic loci in several genes involved in vitamin B12 metabolism have been identified.

**Objective:**

The objective of this literature review was to identify and discuss reports of associations between single-nucleotide polymorphisms (SNPs) in vitamin B12 pathway genes and their influence on the circulating levels of vitamin B12.

**Methods:**

Relevant articles were obtained through a literature search on PubMed through to May 2017. An article was included if it examined an association of a SNP with serum or plasma vitamin B12 concentration. Beta coefficients and odds ratios were used to describe the strength of an association, and a *P* < 0.05 was considered as statistically significant. Two reviewers independently evaluated the eligibility for the inclusion criteria and extracted the data.

**Results:**

From 23 studies which fulfilled the selection criteria, 16 studies identified SNPs that showed statistically significant associations with vitamin B12 concentrations. Fifty-nine vitamin B12-related gene polymorphisms associated with vitamin B12 status were identified in total, from the following populations: African American, Brazilian, Canadian, Chinese, Danish, English, European ancestry, Icelandic, Indian, Italian, Latino, Northern Irish, Portuguese and residents of the USA.

**Conclusion:**

Overall, the data analyzed suggests that ethnic-specific associations are involved in the genetic determination of vitamin B12 concentrations. However, despite recent success in genetic studies, the majority of identified genes that could explain variation in vitamin B12 concentrations were from Caucasian populations. Further research utilizing larger sample sizes of non-Caucasian populations is necessary in order to better understand these ethnic-specific associations.

## Background

Vitamin B12, also known as cobalamin (Cbl), is an essential water-soluble micronutrient required to be ingested by humans to maintain health. The nutritional deficiency of vitamin B12 has been linked to many complications including an increased risk of macrocytic anaemia, neuropsychiatric symptoms [1], cardiovascular diseases [2] and the onset of different forms of cancer [3, 4]. To maintain adequate vitamin B12 status, individuals must ingest sufficient dietary vitamin B12 and retain the ability to absorb vitamin B12. The absorption, transport and cellular uptake of vitamin B12 is dependent upon the co-ordinated action of the binding proteins: haptocorrin (HC), intrinsic factor (IF), transcobalamin II (TC) and other specific cell receptors. After vitamin B12 binds to HC in the stomach and IF in the duodenum, it binds to TC within the enterocyte and is then released into the blood stream. The vitamin B12-TC complex then binds to the transcobalamin receptor (TC-R) and is taken up by cells via endocytosis [5].

Genetic variants may alter vitamin B12 tissue status by affecting the proteins involved in vitamin B12 absorption, cellular uptake and intracellular metabolism [6]. In a study using monozygotic and dizygotic twins, the heritability of B12 levels was estimated to be 59%, indicating that the magnitude of genetic influence on vitamin B12 levels are considerable [7]. At present, genetic studies of vitamin B12 status suggest that it is a multifactorial trait, where several single-nucleotide polymorphisms (SNPs) in multiple genes interact with the environment to cause the altered B12 status [8]. Most of the SNPs related to vitamin B12 status have been examined using a candidate gene approach [8]. However, it is now possible to use an unbiased genome-wide association (GWA) study to associate DNA sequence variations across the human genome with the risk factors of developing a disease [9]. Despite a number of informative genome-wide association studies and candidate gene analyses, the complex relationship between an individual’s genotype and their vitamin B12 status remains poorly understood. This article is the first literature review to discuss the results of genetic studies associated with vitamin B12 status in healthy individuals. Understanding the possible underlying genetic factors of vitamin B12 metabolism will lead to an increased understanding of the biological mechanisms underlying vitamin B12 status.

## Materials and methods

### Study identification

In order to identify published articles, literature searches were completed using the PubMed database (https://www.ncbi.nlm.nih.gov/pubmed/), from the earliest date of indexing until May 2017. The following keywords were used to identify articles from PubMed: ‘vitamin B12 and genetics’ (*n* = 2792), ‘vitamin B12 and gene polymorphisms’ (*n* = 447), ‘genetic variants of vitamin B12’ (*n* = 115), ‘genetic variants of cobalamin’ (*n* = 95), ‘genetics of cobalamin’ (*n* = 2574), ‘genetics of vitamin B12’(*n* = 2721) ‘vitamin B12 and genes’ (*n* = 932) and ‘cobalamin and genes’ (*n* = 858). In addition, reference lists of identified publications were hand searched to identify other studies potentially eligible for inclusion.

No limits on geographical location were placed in the literature search, and only articles written in English were selected. After inclusion and exclusion criteria were applied, a comprehensive list of relevant articles was included in this review.

### Study selection

The abstracts of all articles with relevant titles were reviewed first and were further assessed if they reported original data on testing for an association of a SNP with plasma or serum vitamin B12 concentrations. Articles were excluded if (1) they included non-human subjects, (2) they were limited to a subset of the population (e.g. pregnant women/carrying a disease) and (3) the sample size of the population was less than 10.

**Fig. 1:**
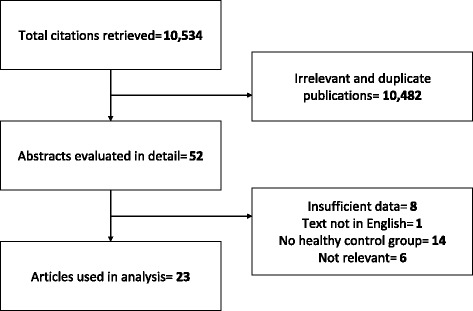
Flow diagram of studies identified in the literature search for the identification of genetic variants associated with vitamin B12 concentrations

Based on the search criteria and keywords used, 10,534 articles were identified from the PubMed database. Following this, 10,482 articles were excluded according to the established exclusion criteria, and 52 articles were then considered as potentially relevant for the review. The full text of the 52 articles was read, which resulted in the exclusion of a further 29 articles. As a result, only 23 articles were selected for analysis (Fig. 1). A *P* < 0.05 was considered as statistically significant.

#### Data extraction

The studies were identified by a single investigator (SS), and the following data were double-extracted independently by two reviewers (VKS and IAS): first author, publication year, location or ethnicity of participants, sample size, mean age, study design, SNP position, name and rs ID, genotype and allele distribution by vitamin B12 status. For the outcome data, the beta coefficients of vitamin B12 concentrations per risk allele, odds ratios (ORs) with their corresponding 95% confidence intervals (95% CIs) were extracted. Any discrepancies over extracted data were settled through discussion between the two independent reviewers (VKS and IAS). Finally, corresponding authors were contacted to provide any additional information where needed.

### Results of database search: genes associated with vitamin B12 status

**Fig. 2:**
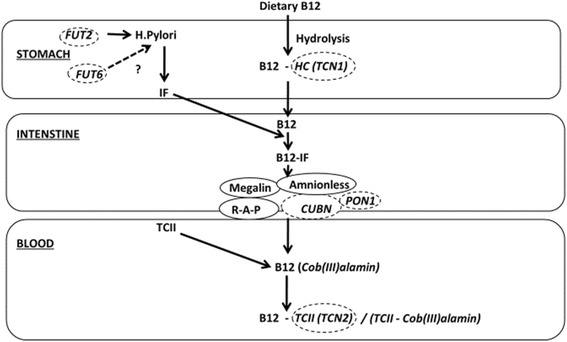
Diagram representing the genes associated with vitamin B12 status. The diagram shows the proteins involved in the metabolism of vitamin B12 from dietary intake to reaching the circulatory system. Genes identified to harbour variants regulating serum levels of B12 are surrounded by dashed lines. B12 vitamin B12, CUBN cubilin (intrinsic factor-cobalamin receptor), FUT2 fucosyl-transferase 2, FUT6 fucosyl-transferase 6, HC haptocorrin (TCN1), *H. pylori Helicobacter pylori*, IF intrinsic factor, PON1 serum paraoxonase/arylesterase 1, R-A-P receptor-associated-protein, TCII transcobalamin II (TCN2), TCII-R transcobalamin II receptor (CD320)

**Fig. 3:**
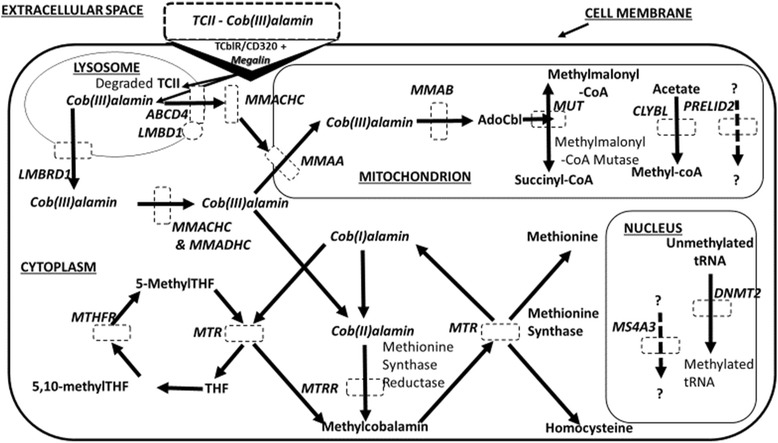
Diagram representing the genes associated with vitamin B12 status. The diagram shows the proteins involved in the metabolism of vitamin B12 from the extracellular space to being internalized within the cell. Genes identified to harbour variants regulating serum levels of B12 are surrounded by dashed lines. Ado-B12 adenosyl-cobalamin; ABDC4 ATP-binding cassette, sub-family D (ALD), member 4; CD320 CD320 molecule; CLYBL citrate lyase beta like; DNMT2 DNA methyltransferase 2 gene; LMBD1 LMBR1 domain containing 1; LMBRD1 LMBR1 domain containing 1; MMAA methylmalonic aciduria (cobalamin deficiency) CblA type; MMAB methylmalonic aciduria (cobalamin deficiency) CblB type; MMACHC methylmalonic aciduria and homocystinuria, cblC type; MMADHC methylmalonic aciduria (cobalamin deficiency) CblD type, with homocystinuria; MS4A3 membrane-spanning 4-domains, subfamily A, member 3 (hematopoietic cell-specific); MTHFR 5-methyl-tetrahydrafolate reductase; MTR 5-methyltetrahydrofolate-homocysteine methyltransferase; MTRR 5-methyltetrahydrofolate-homocysteine methyltransferase reductase; MUT methylmalonyl CoA mutase; PRELID2 PRELI domain containing 2; THF tetrahydrofolate; 5,10-Methyl THF 5,10-methyl-tetrahydrofolate

The following section reviews studies of genetic variants which have been associated with vitamin B12 status. These variants have been grouped as (a) co-factors or regulators essential for the transport of vitamin B12, (b) membrane transporters actively facilitating membrane crossing (c) involved in the catalysis of enzymatic reactions in the one carbon cycle (d) involved in cell cycle regulation, (e) mitochondrial proteins and (f) other genes (Figs. 2 and 3). A summary of GWA and candidate gene association studies that have been reported to be associated with circulating plasma or serum B12 concentrations are presented in Table 1 and Table 2. The location and function of the most frequently studied genes associated with vitamin B12 concentrations are summarized in Table 3.

**Table 1:** Genome-wide association studies showing the association of SNPs with vitamin B12 concentrations. Genome-wide association studies testing the association between SNPs and vitamin B12 concentrations. The chromosome location, gene name, reference SNP cluster ID, sample size and ethnicity, mean age, observed frequency of the minor allele in the population, effect size and *P* value are shown in the table. The order of SNPs reflects the order of the chromosome location

Chromosome location	Gene name (gene symbol)	Reference SNP cluster ID	Sample size and ethnicity	Age (years)	Minor allele + minor allele frequency	Effect size	*P* value	References
1p34.1	Methylmalonic aciduria and homocystinuria type C protein (*MMACHC*)	rs12272669	Icelandic sample: *n* = 37,283	63 ± 24	A = 0.002	Effect: A allele Other: G allele *β* = 0.51 pmol/l	3.00 × 10^−9^	Grarup et al. [12]
1q42.2	Intergenic	rs583228	Initial sample: *n* = 1999 Chinese Han men	38 ± 11	*T* = 0.220	Effect: T allele Other: C allele *β* = not available	7.68 × 10^−6^	Lin et al. [19]
			Replication sample: *n* = 1496 Chinese men	37 ± 11		Effect: T allele Other: C allele *β* = not available	> 0.05	
			Combined total: *n* = 3495			Effect: T allele Other: C allele *β* = 25.50 pg/ml SE = 7.19	3.92 × 10^−4^	
2q34	Carbamoyl-phosphate synthase 1 (*CPS1*)	rs1047891	Icelandic sample: *n* = 37,283	63 ± 24	A = 0.372	Effect: C allele Other: A allele *β* = 0.04 pmol/l	7.60 × 10^−6^	Grarup et al. [12]
			Danish Inter99 population: *n* = 5481	46 ± 8		Effect: C allele Other: A allele *β* = 0.10 pmol/l	5.50 × 10^−4^	
			Danish Health 2006: *n* = 2812	49 ± 13		Effect: C allele Other: A allele *β* = 0.03 pmol/l	> 0.05	
			Combined total: *n* = 45,574			Effect: C allele Other: A allele *β* = not available	3.00 × 10^−8^	
4q31.21	Methylmalonic aciduria (cobalamin deficiency) cblA type (*MMAA*)	rs2270655	Parents of PMNS cohort^*^: *n* = 1001 Indian	36 ± 5	C = 0.157^#^	Effect allele: C *β* = − 0.07 pmol/l	> 0.05	Nongmaithem et al. [22]
			adults: *n* = 724 Indian	38 ± 11		Effect allele: C *β* = 0.00 pmol/l	> 0.05	
			PMNS children^*^: *n* = 690 Indian	11 ± 1		Effect allele: C *β* = − 0.09 pmol/l	> 0.05	
			PS children^†^: *n* = 534 Indian	5 ± 0		Effect allele: C *β* = − 0.20 pmol/l	2.00 × 10^−2^	
4q31.21	Methylmalonic aciduria (cobalamin deficiency) cblA type (*MMAA*)	rs2270655	Icelandic sample: *n* = 37,283	63 ± 24	C = 0.059	Effect: G allele Other: C allele *β* = 0.07 pmol/l	3.50 × 10^−5^	Grarup et al. [12]
			Danish Inter99 population: *n* = 5481	46 ± 8		Effect: G allele Other: C allele *β* = 0.30 pmol/l	2.80 × 10^−7^	
			Danish Health 2006: *n* = 2812	49 ± 13		Effect: G allele Other: C allele *β* = 0.25 pmol/l	5.80 × 10^−8^	
			Combined total: *n* = 45,576			Effect: G allele Other: C allele *β* = not available	2.20 × 10^−13^	
4q31.21	Methylmalonic aciduria (cobalamin deficiency) cblA type (*MMAA*)	rs114699496	Icelandic sample: *n* = 25,960	63 ± 24	*T* = 0.046^**^	Effect: T Other: C *β* = − 0.07 pmol/l	7.60 × 10^−6^	Grarup et al. [12]
5q32	Intergenic	rs10515552	Initial sample: *n* = 1999 Chinese Han men	38 ± 11	C = 0.162	Effect: C allele Other: T allele *β* = not available	8.52 × 10^−7^	Lin et al. [19]
			Replication sample: *n* = 1496 Chinese men	37 ± 11		Effect: C allele Other: T allele *β* = not available	5.15 × 10^−3^	
			Combined total: *n* = 3495			Effect: C allele Other: T allele *β* = 43.93 pg/ml SE = 7.98	3.94 × 10^−8^	
6p12.3	Methylmalonyl-CoA Mutase (*MUT*)	chr6:49,508,102	Icelandic sample: *n* = 25,960	63 ± 24	Not available	Effect: C allele Other: G allele *β* = 0.07 pmol/l	1.60 × 10^−18^	Grarup et al. [12]
6p12.3	Methylmalonyl-CoA Mutase (*MUT*)	rs1141321 (rs9473558)	Icelandic sample: *n* = 37,283	63 ± 24	*T* = 0.373	Effect: C allele Other: T allele *β* = 0.06 pmol/l	1.40 × 10^−16^	Grarup et al. [12]
			Danish Inter99 population: *n* = 5481	46 ± 8		Effect: C allele Other: T allele *β* = 0.12 pmol/l	1.40 × 10^−5^	
			Danish Health 2006: *n* = 2812	49 ± 13		Effect: C allele Other: T allele *β* = 0.11 pmol/l	1.40 × 10^−7^	
			Combined total: *n* = 45,574			Effect: C allele Other: T allele *β* = not available	3.60 × 10^−26^	
6p12.3	Methylmalonyl-CoA mutase (*MUT*)	rs1141321 (rs9473558)	Initial sample: *n* = 1999 Chinese Han men	38 ± 11	*T* = 0.237	Effect: T allele Other: C allele *β* = − 30.34 pg/ml SE = 8.91	5.51 × 10^−4^	Lin et al. [19]
6p12.3	Methylmalonyl-CoA mutase (*MUT*)	rs1141321 (rs9473558)	NHS-CGEMS^‡^: *n* = 1658 Caucasian women	59 ± 6	*T* = 0.350	Effect: T allele Other: C allele *β* = − 0.03 pg/ml SE = 0.01	4.27 × 10^−2^	Hazra et al. [20]
			SHARe^§^: *n* = 1647 Caucasian women	59 ± 10		Effect: T allele Other: C allele *β* = − 0.03 pg/ml SE = 0.01	1.87 × 10^−2^	
			SHARe^§^: *n* = 1458 Caucasian men	59 ± 10		Effect: T allele Other: C allele *β* = − 0.07 pg/ml SE = 0.01	3.96 × 10^−7^	
			Combined total: *n* = 4763			Effect: T allele Other: C allele *β* = − 0.04 pg/ml SE = 0.01	4.05 × 10^−8^	
6p12.3	Methylmalonyl-CoA mutase (*MUT*)	rs9473555	Icelandic sample: *n* = 25,960	63 ± 24	C = 0.402	Effect: C allele Other: G allele *β* = − 0.06 pmol/l	5.40 × 10^−17^	Grarup et al. [12]
6p12.3	Methylmalonyl-CoA mutase (*MUT*)	rs9473555	Initial sample: *n* = 1999 Chinese Han men	38 ± 11	C = 0.238	Effect: C allele Other: G allele *β* = − 31.00 pg/ml SE = 8.860	4.06 × 10^−4^	Lin et al. [19]
6p12.3	Methylmalonyl-CoA mutase (*MUT*)	rs9473555	NHS-CGEMS^‡^: *n* = 1658 Caucasian women	59 ± 6	C = 0.350	Effect: C allele Other: G allele *β* = − 0.03 pg/ml SE = 0.01	4.27 × 10^−2^	Hazra et al. [20]
			SHARe^§^: *n* = 1647 Caucasian women	59 ± 10		Effect: C allele Other: G allele *β* = − 0.03 pg/ml SE = 0.01	2.26 × 10^−2^	
			SHARe^§^: *n* = 1458 Caucasian men	59 ± 10		Effect: C allele Other: G allele *β* = − 0.07 pg/ml SE = 0.01	3.71 × 10^−7^	
			Combined total: *n* = 4763			Effect: C allele Other: G allele *β* = − 0.04 pg/ml SE = 0.01	4.91 × 10^−8^	
6q15	Nearest gene: sperm acrosome associated 1 (*SPACA1*)	Chr6_88,792,234	Icelandic sample: *n* = 37,283	63 ± 24	G = 0.006	Effect: G allele Other: A allele *β* = 0.26 pmol/l	2.80 × 10^−7^	Grarup et al. [12]
7q21.3	Paraoxonase 1 (*PON1*)	rs3917577	*n* = 3114 Canadian (85% Causasian, 15% non-Caucasian)	20–79 (range)	G = 0.020	Effect:A allele Other: G allele Vitamin B-12 below adequate (< 220 pmol/l): OR 0.67 (95% CI 0.54, 0.81) pmol/l	7.20 × 10^−5^	Zinck et al. [18]
8q21.13	Nearest gene: zinc finger and BTB domain containing 10 (*ZBTB10*)	rs62515066	Icelandic sample: *n* = 37,283	63 ± 24	G = 0.025	Effect: G allele Other: A allele *β* = 0.12 pmol/l	5.40 × 10^−7^	Grarup et al. [12]
9p21.1	None (Intergenic)	rs12377462	Initial sample: *n* = 1999 Chinese Han men	38 ± 11	*T* = 0.366	Effect: T allele Other: C allele *β* = not available	3.34 × 10^−7^	Lin et al. [19]
			Replication sample: *n* = 1496 Chinese men	37 ± 11		Effect: T allele Other: C allele *β* = not available	> 0.05	
			Combined total: *n* = 3495			Effect: T allele Other: C allele *β* = 28.53 pg/ml SE = 5.99	2.02 × 10^−6^	
10p12.31	Cubulin (*CUBN)*	rs1801222	*n* = 3114 Canadian (85% Causasian, 15% non-Caucasian)	20–79 (range)	A = 0.100	Effect: G allele Other: A allele Vitamin B12 deficiency (< 148 pmol/l): OR 1.61 (95% CI 1.24, 2.09) pmol/l	3.00 × 10^−4^	Zinck et al. [18]
10p12.31	Cubulin (*CUBN)*	rs1801222	*n* = 3114 Canadian (85% Causasian, 15% non-Caucasian)	20–79 (range)	A = 0.100	Effect: G allele Other: A allele Vitamin B-12 below adequate (< 220 pmol/l): OR 1.39 (95% CI 1.23, 1.58) pmol/l	2.00 × 10^−7^	Zinck et al. [18]
10p12.31	Cubulin (*CUBN)*	rs1801222	Icelandic sample: *n* = 37,283	63 ± 24	A = 0.407	Effect: G allele Other: A allele *β* = 0.10 pmol/l	1.10 × 10^−52^	Grarup et al. [12]
			Danish Inter99 population: *n* = 5481	46 ± 8		Effect: G allele Other: A allele *β* = 0.14 pmol/l	7.60 × 10^−8^	
			Danish Health 2006: *n* = 2812	49 ± 13		Effect: G allele Other: A allele *β* = 0.17 pmol/l	2.90 × 10^−18^	
			Combined total: *n* = 45,576			Effect: G allele Other: A allele *β* = not available	3.30 × 10^−75^	
10p12.31	Cubulin (*CUBN)*	rs1801222	NHS-CGEMS^‡^: *n* = 1658 Caucasian women	59 ± 6	A = 0.280	Effect: A allele Other: G allele *β* = − 0.05 pg/ml SE = 0.01	9.04 × 10^−5^	Hazra et al. [20]
			SHARe^§^: *n* = 1647 Caucasian women	59 ± 10		Effect: A allele Other: G allele *β* = − 0.04 pg/ml SE = 0.02	6.32 × 10^−3^	
			SHARe^§^: *n* = 1458 Caucasian men	59 ± 10		Effect: A allele Other: G allele *β* = − 0.05 pg/ml SE = 0.02	3.56 × 10^−4^	
			Combined total: *n* = 4763			Effect: A allele Other: G allele *β* = − 0.05 pg/ml SE = 0.01	2.87 × 10^−9^	
10p12.31	Cubulin (*CUBN)*	rs4748353	*n* = 3114 Canadian (85% Causasian, 15% non-Caucasian)	20–79 (range)	C = 0.000	Effect: C allele Other: T allele Vitamin B12 deficiency (< 148 pmol/l): OR 2.14 (95% CI 1.36, 3.37) pmol/l	8.00 × 10^−4^	Zinck et al. [18]
10p12.31	Cubulin (*CUBN)*	rs11254363	*n* = 3114 Canadian (85% Causasian, 15% non-Caucasian)	20 – 79 (range)	G = 0.010	Effect: A allele Other: G allele Vitamin B-12 below adequate (< 220 pmol/l): OR 0.81 (95% CI 0.70, 0.93) pmol/l	3.00 × 10^−3^	Zinck et al. [18]
10p12.31	Cubulin (*CUBN)*	rs11254363	GWAS Meta-analysis: InCHIANTI study: *n* = 1175 Italian SardiNIA study: *n* = 1115 Italian BLSA study^¶^: *n* = 640 Residents from the USA	InCHIANTI: 68 ± 16 SardiNIA: 45 ± 18 BLSA^g^: 68 ± 16	G = 0.300	Effect: A allele Other: G allele *β* = − 39.16 pg/ml SE = 9.18	7.24 × 10^−8^	Tanaka et al. [21]
			Replication study: Progetto Nutrizione study: *n* = 687 Italian	47 ± 13		Effect: A allele Other: G allele *β* = 3.62 pg/ml SE = 10.94	> 0.05	
			Combined meta-analysis (GWAS Meta-analysis + Replication study): *n* = 3613			Effect: A allele Other: G allele *β* = − 21.49 pg/ml SE = 7.03	1.11 × 10^−6^	
10p12.31	Cubulin (*CUBN)*	rs12243895	Initial sample: *n* = 1999 Chinese Han men	38 ± 11	A = 0.243	Effect: A allele Other: G allele *β* = 23.49 pg/ml SE = 9.06	7.11 × 10^−3^	Lin et al. [19]
10p12.31	Cubulin (*CUBN)*	rs12780845	Parents of PMNS cohort^*^: *n* = 1001 Indian	36 ± 5	G = 0.415^#^	Effect allele: G *β* = 0.09 pmol/l	> 0.05	Nongmaithem et al. [22]
			Adults: *n* = 724 Indian	38 ± 11		Effect allele: G *β* = 0.09 pmol/l	> 0.05	
			PMNS children^*^: *n* = 690 Indian	11 ± 1		Effect allele: G *β* = 0.08 pmol/l	> 0.05	
			PS children^†^: *n* = 534 Indian	5 ± 0		Effect allele: G *β* = 0.03 pmol/l	> 0.05	
10p13	DNA methyltransferase gene (*DNMT2*)/TRNA aspartic acid methyltransferase 1 (*TRDMT1*)	rs2295809	*n* = 3114 Canadian (85% Causasian, 15% non-Caucasian)	20–79 (range)	*T* = 0.240	Effect: A allele Other: T allele Vitamin B-12 below adequate (< 220 pmol/l): OR 0.82 (95% CI 0.73, 0.92) pmol/l	1.00 × 10^−3^	Zinck et al. [18]
10p13	DNA methyltransferase gene (*DNMT2*)/TRNA aspartic acid methyltransferase 1 (*TRDMT1*)	rs56077122	Icelandic sample: *n* = 25,960	63 ± 24	A = 0.335	Effect: A allele Other: C allele *β* = 0.09 pmol/l	4.80 × 10^−21^	Grarup et al. [12]
11q12.1	Intergenic Nearest gene: transcobalamin 1 (*TCN1*)	rs117456053	Icelandic sample: *n* = 25,960	63 ± 24	A = 0.024	Effect: G allele Other: A allele *β* = 0.16 pmol/l	1.90 × 10^−9^	Grarup et al. [12]
11q12.1	Membrane Spanning 4-Domains A3 *(MS4A3)*	rs2298585	Icelandic sample: *n* = 25,960	63 ± 24	*T* = 0.001	Effect: T allele Other: C allele *β* = 0.21 pmol/l	> 0.05	Grarup et al. [12]
11q12.1	Membrane Spanning 4-Domains A3 *(MS4A3)*	rs2298585	Initial sample: *n* = 1999 Chinese Han men	38 ± 11	*T* = 0.120	Effect: T allele Other: C allele *β* = not available	1.71 × 10^−10^	Lin et al. [19]
			Replication sample: *n* = 1496 Chinese men	37 ± 11		Effect: T allele Other: C allele *β* = not available	1.58 × 10^−6^	
			Combined total: *n* = 3495			Effect: T allele Other: C allele *β* = 71.80 pg/ml SE = 9.04	2.64 × 10^−15^	
11q12.1	Transcobalamin 1 (*TCN1*)	rs526934	Adults: *n* = 724 Indian	38 ± 11	G = 0.216^#^	Effect allele: G *β* = − 0.07 pmol/l	> 0.05	Nongmaithem et al. [22]
			PMNS children^*^: *n* = 690 Indian	11 ± 1		Effect allele: G *β* = − 0.10 pmol/l	> 0.05	
			PS children^†^: *n* = 534 Indian	5 ± 0		Effect allele: G *β* = − 0.16 pmol/l	2.00 × 10^−2^	
11q12.1	Transcobalamin 1 (*TCN1*)	rs526934	*n* = 3114 Canadian (85% Causasian, 15% non-Caucasian)	20–79 (range)	G = 0.080	Effect: A allele Other: G allele Vitamin B-12 below adequate (< 220 pmol/l): OR 1.38 (95% CI 1.21, 1.57) pmol/l	1.40 × 10^−6^	Zinck et al. [18]
11q12.1	Transcobalamin 1 (*TCN1*)	rs526934	Icelandic sample: *n* = 25,960	63 ± 24	G = 0.296	Effect: G allele Other: A allele *β* = − 0.12 pmol/l	2.30 × 10^−48^	Grarup et al. [12]
11q12.1	Transcobalamin 1 (*TCN1*)	rs526934	Initial sample: *n* = 1999 Chinese Han men	8 ± 11	G = 0.189	Effect: G allele Other: A allele *β* = − 30.39 pg/ml SE = 9.66	1.78 × 10^−3^	Lin et al. [19]
11q12.1	Transcobalamin 1 (*TCN1*)	rs526934	NHS-CGEMS^‡^: *n* = 1658 Caucasian women	59 ± 6	G = 0.270	Effect: G allele Other: A allele *β* = − 0.05 pg/ml SE = 0.01	1.27 × 10^−3^	Hazra et al. [20]
			SHARe^§^: *n* = 1647 Caucasian women	59 ± 10		Effect: G allele Other: A allele *β* = − 0.06 pg/ml SE = 0.02	6.69 × 10^−5^	
			SHARe^§^: *n* = 1458 Caucasian men	59 ± 10		Effect: G allele Other: A allele *β* = − 0.06 pg/ml SE = 0.02	1.64 × 10^−4^	
			Combined total: *n* = 4763			Effect: G allele Other: A allele *β* = − 0.05 pg/ml SE = 0.01	2.25 × 10^−10^	
11q12.1	Transcobalamin 1 (*TCN1*)	rs526934	GWAS Meta-analysis: InCHIANTI study: *n* = 1175 Italian SardiNIA study: *n* = 1115 Italian BLSA study^¶^: *n* = 640 Residents from the USA	InCHIANTI: 68 ± 16 SardiNIA: 45 ± 18 BLSA^g^: 68 ± 16	G = 0.330	Effect: A allele Other: G allele *β* = 36.76 pg/ml SE = 10.35	8.33 × 10^−7^	Tanaka et al. [21]
			Replication study: Progetto Nutrizione study: *n* = 687 Italian	47 ± 14		Effect: A allele Other: G allele *β* = 12.83 pg/ml SE = 13.24	> 0.05	
			Combined meta-analysis (GWAS Meta-analysis + Replication study): *n* = 3613			Effect: A allele Other: G allele *β* = 27.62 pg/ml SE = 8.15	1.51 × 10^−6^	
11q12.1	Transcobalamin 1 (*TCN1*)	rs34324219	Adults: *n* = 724 Indian	38 ± 11	A = 0.041^††^	Effect allele: A *β* = − 0.30 pmol/l	2.00 × 10^−2^	Nongmaithem et al. [22]
			PMNS children^*^: *n* = 690 Indian	11 ± 1		Effect allele: A *β* = − 0.14 pmol/l	> 0.05	
			PS children^†^: *n* = 534 Indian	5 ± 0		Effect allele: A *β* = − 0.65 pmol/l	9.50 × 10^−7^	
11q12.1	Transcobalamin 1 (*TCN1*)	rs34324219	Icelandic sample: *n* = 37,283	63 ± 24	A = 0.119	Effect: C allele Other: A allele *β* = 0.21 pmol/l	8.80 × 10^−71^	Grarup et al. [12]
			Danish Inter99 population: *n* = 5481	46 ± 8		Effect: C allele Other: A allele *β* = 0.40 pmol/l	3.20 × 10^−23^	
			Danish Health 2006: *n* = 2812	49 ± 13		Effect: C allele Other: A allele *β* = 0.30 pmol/l	3.50 × 10^−24^	
			Combined total: *n* = 45,576			Effect: C allele Other: A allele *β* = not available	1.10 × 10^−111^	
11q12.1	Transcobalamin 1 (*TCN1*)	rs34528912	Adults: *n* = 724 Indian	38 ± 11	*T* = 0.006^††^	Effect allele: T *β* = − 0.79 pmol/l	1.00 × 10^−2^	Nongmaithem et al. [22]
			PMNS children^*^: *n* = 690 Indian	11 ± 1		Effect allele: T *β* = 0.38 pmol/l	> 0.05	
			PS children^†^: *n* = 534 Indian	5 ± 0		Effect allele: T *β* = − 0.47 pmol/l	3.00 × 10^−2^	
11q12.1	Transcobalamin 1 (*TCN1*)	rs34528912	Icelandic sample: *n* = 25,960	63 ± 24	*T* = 0.036	Effect: T allele Other: C allele *β* = 0.17 pmol/l	2.10 × 10^−15^	Grarup et al. [12]
13q32.3	Citrate Lyase Beta Like (*CLYBL*)	rs41281112	Initial sample: *n* = 1999 Chinese Han men	38 ± 11	*T* = 0.044	Effect: T allele Other: C allele *β* = not available	1.09 × 10^−8^	Lin et al. [19]
			Replication sample: *n* = 1496 Chinese men	37 ± 11		Effect: T allele Other: C allele *β* = not available	7.41 × 10^−3^	
			Combined total: *n* = 3495			Effect: T allele Other: C allele *β* = − 83.60 pg/ml SE = 13.62	9.23 × 10^−10^	
13q32.3	Citrate Lyase Beta Like (*CLYBL*)	rs41281112	Icelandic sample: *n* = 37,283	63 ± 24	*T* = 0.052	Effect: C allele Other: T allele *β* = 0.17 pmol/l	9.60 × 10^−27^	Grarup et al. [12]
			Danish Inter99 population: *n* = 5481	46 ± 8		Effect: C allele Other: T allele *β* = 0.24 pmol/l	1.30 × 10^−3^	
			Danish Health 2006: *n* = 2812	49 ± 13		Effect: C allele Other: T allele *β* = 0.29 pmol/l	2.50 × 10^−7^	
			Combined total: *n* = 45,576			Effect: C allele Other: T allele *β* = not available	8.90 × 10^−35^	
14q24.3	ATP Binding Cassette Subfamily D Member 4 (*ABCD4*)	rs3742801	Icelandic sample: *n* = 37,283	63 ± 24	*T* = 0.294	Effect: T allele Other: C allele *β* = 0.05 pmol/l	5.30 × 10^−8^	Grarup et al. [12]
			Danish Inter99 population: *n* = 5481	46 ± 8		Effect: T allele Other: C allele *β* = 0.09 pmol/l	7.60 × 10^−4^	
			Danish Health 2006: *n* = 2812	49 ± 13		Effect: T allele Other: C allele *β* = 0.08 pmol/l	4.50 × 10^−5^	
			Combined total: *n* = 45,571			Effect: T allele Other: C allele *β* = not available	1.70 × 10^−13^	
14q24.3	ATP binding cassette subfamily D member 4 (*ABCD4*)	rs4619337	Icelandic sample: *n* = 25,960	63 ± 24	C = 0.292^‡‡^	Effect: C allele Other: T allele *β* = 0.05 pmol/l	3.40 × 10^−8^	Grarup et al. [12]
19p13.2	Actin like 9 (*ACTL9*)	rs2340550	Initial sample: *n* = 1999 Chinese Han men	38 ± 11	A = 0.134	Effect: A allele Other: G allele *β* = not available	9.34 × 10^−7^	Lin et al. [19]
			Replication sample: *n* = 1496 Chinese men	37 ± 11		Effect: A allele Other: G allele *β* = not available	> 0.05	
			Combined total: *n* = 3495			Effect: A allele Other: G allele *β* = 23.39 pg/ml SE = 8.56	6.32 × 10^−3^	
19p13.2	CD320 molecule (*CD320*)/transcobalamin II receptor (*TcblR*)	rs2336573	*n* = 3114 Canadian (85% Causasian, 15% non-Caucasian)	20–79 (range)	*T* = 0.010	Effect: C allele Other: T allele Vitamin B-12 below adequate (< 220 pmol/l): OR 0.62 (95% CI 0.45, 0.86) pmol/l	3.0 × 10^−3^	Zinck et al. [18]
19p13.2	CD320 molecule (*CD320*) / Transcobalamin II Receptor (*TcblR*)	rs2336573	Icelandic sample: *n* = 37,283	63 ± 24	*T* = 0.031	Effect: T allele Other: C allele *β* = 0.32 pmol/l	1.10 × 10^−51^	Grarup et al. [12]
			Danish Inter99 population: *n* = 5481	46 ± 8		Effect: T allele Other: C allele *β* = 0.22 pmol/l	5.70 × 10^−3^	
			Danish Health 2006: *n* = 2812	49 ± 13		Effect: T allele Other: C allele *β* = 0.31 pmol/l	1.70 × 10^−8^	
			Combined total: *n* = 45,575			Effect: T allele Other: C allele *β* = not available	8.40 × 10^−59^	
19p13.2	CD320 molecule (*CD320*) / Transcobalamin II receptor (*TcblR*)	rs8109720	Icelandic sample: *n* = 25,960	63 ± 24	Not available	Effect: G allele Other: A allele *β* = 0.32 pmol/l	5.80 × 10^−52^	Grarup et al. [12]
19q13.33	Fucosyl transferase 2 gene (*FUT2*)	rs281379	Parents of PMNS cohort^*^: *n* = 1001 Indian	36 ± 5	A = 0.222^#^	Effect allele: A *β* = 0.20 pmol/l	4.60 × 10^−4^	Nongmaithem et al. [22]
			Adults: *n* = 724 Indian	38 ± 11		Effect allele: A *β* = 0.05 pmol/l	> 0.05	
			PMNS children^*^: *n* = 690 Indian	11 ± 1		Effect allele: A *β* = 0.24 pmol/l	4.50 × 10^−4^	
			PS children^†^: *n* = 534 Indian	5 ± 0		Effect allele: A *β* = 0.13 pmol/l	> 0.05	
19q13.33	Fucosyl transferase 2 gene (*FUT2*)	rs492602	*n* = 3114 Canadian (85% Causasian, 15% non-Caucasian)	20–79 (range)	A = 0.210	Effect: G allele Other: A allele Vitamin B12 deficiency (< 148 pmol/l): OR 0.60 (95% CI 0.54, 0.70) pmol/l	2.00 × 10^−4^	Zinck et al. [18]
19q13.33	Fucosyl transferase 2 gene (*FUT2*)	rs492602	*n* = 3114 Canadian (85% Causasian, 15% non-Caucasian)	20–79 (range)	A = 0.210	Effect: G allele Other: A allele Vitamin B-12 below adequate (< 220 pmol/l): OR 0.71 (95% CI 0.65, 0.81) pmol/l	9.00 × 10^−8^	Zinck et al. [18]
19q13.33	Fucosyl transferase 2 gene (*FUT2*)	rs492602	NHS-CGEMS^‡^: *n* = 1658 Caucasian women	59 ± 6	G = 0.440	Effect: G allele Other: A allele *β* = 0.09 pg/ml SE = 0.01	5.39 × 10^−11^	Hazra et al. [20]
			SHARe^§^: *n* = 1647 Caucasian women	59 ± 10		Effect: G allele Other: A allele *β* = 0.04 pg/ml SE = 0.02	5.89 × 10^−3^	
			SHARe^§^: *n* = 1458 Caucasian men	59 ± 10		Effect: G allele Other: A allele *β* = 0.05 pg/ml SE = 0.01	2.36 × 10^−4^	
			Combined total: *n* = 4763			Effect: G allele Other: A allele *β* = 0.06 pg/ml SE = 0.01	1.30 × 10^−14^	
19q13.33	Fucosyl transferase 2 gene (*FUT2*)	rs492602	NHS-CGEMS^‡^: *n* = 1637 Caucasian women	59 (Mean)	G = 0.490	Effect: A allele Other: G allele *β* = − 0.08 pg/ml SE = 0.01	2.68 × 10^−10^	Hazra et al. [29]
			Replication: *n* = 1059 Caucasian women	63 (Mean)		Effect: A allele Other: G allele *β* = − 0.10 pg/ml SE = 0.02	5.60 × 10^−9^	
			Combined meta-analysis: *n* = 2696			Effect: A allele Other: G allele *β* = − 0.09 pg/ml SE = 0.01	5.36 × 10^−17^	
19q13.33	Fucosyl transferase 2 gene (*FUT2*)	rs516316	Icelandic sample: *n* = 25,960	63 ± 24	C = 0.469^‡‡^	Effect: C allele Other: G allele *β* = 0.17 pmol/l	3.60 × 10^−103^	Grarup et al. [12]
19q13.33	Fucosyl transferase 2 gene (*FUT2*)	rs601338	Adults: *n* = 724 Indian	38 ± 11	A = 0.230^#^	Effect: A Other: G *β* = 0.05 pmol/l	> 0.05	Nongmaithem et al. [22]
			PMNS children^*^: *n* = 690 Indian	11 ± 1		Effect: A Other: G *β* = 0.25 pmol/l	3.8 × 10^−5^	
			PS children^†^: *n* = 534 Indian	5 ± 0		Effect: A Other: G *β* = 0.18 pmol/l	4.30 × 10^−3^	
19q13.33	Fucosyl transferase 2 gene (*FUT2*)	rs601338	*n* = 25,960 Icelandic	63 ± 24	G = 0.384	Effect: G allele Other: A allele *β* = − 0.16 pmol/l	2.40 × 10^−95^	Grarup et al. [12]
19q13.33	Fucosyl transferase 2 gene (*FUT2*)	rs601338	NHS-CGEMS^‡^: *n* = 1658 Caucasian women	59 ± 6	A = 0.450	Effect: A allele Other: G allele *β* = 0.09 pg/ml SE = 0.01	4.25 × 10^−11^	Hazra et al. [20]
			SHARe^§^: *n* = 1647 Caucasian women	59 ± 10		Effect: A allele Other: G allele *β* = 0.05 pg/ml SE = 0.01	2.63 × 10^−3^	
			SHARe^§^: *n* = 1458 Caucasian men	59 ± 10		Effect: A allele Other: G allele *β* = 0.05 pg/ml SE = 0.01	4.02 × 10^−4^	
			Combined total: *n* = 4763			Effect: A allele Other: G allele *β* = 0.06 pg/ml SE = 0.01	6.92 × 10^−15^	
19q13.33	Fucosyl transferase 2 gene (*FUT2*)	rs601338	NHS-CGEMS^‡^: *n* = 1658 Caucasian women	59 (Mean)	G = 0.490	Effect: G allele Other: A allele *β* = − 0.08 pg/ml SE = 0.01	4.11 × 10^−10^	Hazra et al. [29]
19q13.33	Fucosyl transferase 2 gene (*FUT2*)	rs602662	Adults: *n* = 724 Indian	38 ± 11	A = 0.233^#^	Effect allele: A *β* = 0.10 pmol/l	> 0.05	Nongmaithem et al. [22]
			PMNS children^*^: *n* = 690 Indian	11 ± 1		Effect allele: A *β* = 0.25 pmol/l	1.90 × 10^−5^	
			PS children^†^: *n* = 534 Indian	5 ± 0		Effect allele: A *β* = 0.20 pmol/l	1.40 × 10^−3^	
19q13.33	Fucosyl transferase 2 gene (*FUT2*)	rs602662	*n* = 3114 Canadian (85% Causasian, 15% non-Caucasian)	20–79 (range)	G = 0.230	Effect: A allele Other: G allele Vitamin B12 deficiency (< 148 pmol/l): OR 0.61 (95% CI 0.47, 0.80) pmol/l	3.00 × 10^−4^	Zinck et al. [18]
19q13.33	Fucosyl transferase 2 gene (*FUT2*)	rs602662	*n* = 3114 Canadian (85% Causasian, 15% non-Caucasian)	20–79 (range)	G = 0.230	Effect: A allele Other: G allele Vitamin B-12 below adequate (< 220 pmol/l): OR 0.74 (95% CI 0.66, 0.84) pmol/l	1.20 × 10^−6^	Zinck et al. [18]
19q13.33	Fucosyl transferase 2 gene (*FUT2*)	rs602662	Icelandic sample: *n* = 37,283	63 ± 24	G = 0.404	Effect: A allele Other: G allele *β* = 0.16 pmol/l	4.10 × 10^−96^	Grarup et al. [12]
			Danish Inter99 population: *n* = 5481	46 ± 8		Effect: A allele Other: G allele *β* = 0.19 pmol/l	3.50 × 10^−13^	
			Danish - Health 2006: *n* = 2812	49 ± 13		Effect: A allele Other: G allele *β* = 0.23 pmol/l	1.90 × 10^−34^	
			Combined total *n* = 45,568			Effect: A allele Other: G allele *β* = not available	2.40 × 10^−139^	
19q13.33	Fucosyl transferase 2 gene (*FUT2*)	rs602662	NHS-CGEMS^‡^: *n* = 1658 Caucasian women	59 ± 6	G = 0.440	Effect: G allele Other: A allele *β* = − 0.08 pg/ml SE = 0.01	3.09 × 10^−10^	Hazra et al. [20]
			SHARe^§^: *n* = 1647 Caucasian women	59 ± 10		Effect: G allele Other: A allele *β* = − 0.05 pg/ml SE = 0.02	3.80 × 10^−4^	
			SHARe^§^: *n* = 1458 Caucasian men	59 ± 10		Effect: G allele Other: A allele *β* = − 0.05 pg/ml SE = 0.01	2.80 × 10^−4^	
			Combined total: *n* = 4763			Effect: G allele Other: A allele *β* = − 0.07 pg/ml SE = 0.01	1.83 × 10^−15^	
19q13.33	Fucosyl transferase 2 gene (*FUT2*)	rs602662	GWAS Meta-analysis: InCHIANTI study: *n* = 1175 Italian SardiNIA study: *n* = 1115 Italian BLSA study^¶^: *n* = 640 Residents from the USA	InCHIANTI: 68 ± 16 SardiNIA: 45 ± 18 BLSA^g^: 68 ± 16	G = 0.470	Effect: A allele Other: G allele *β* = 44.20 pg/ml SE = 8.26	2.43 × 10^−12^	Tanaka et al. [21]
			Replication study: Progetto Nutrizione study: N = 687 Italian	47 ± 13		Effect: A allele Other: G allele *β* = 58.65 pg/ml SE = 10.43	2.19 × 10^−10^	
			Combined meta-analysis (GWAS Meta-analysis + Replication study): *n* = 3613			Effect: A allele Other: G allele *β* = 49.77 pg/ml SE = 6.47	2.83 × 10^−20^	
19q13.33	Fucosyl transferase 2 gene (*FUT2*)	rs602662	NHS-CGEMS^‡^: *n* = 1658 Caucasian women	59 (Mean)	G = 0.490	Effect: G allele Other: A allele *β* = − 0.08 pg/ml SE = 0.01	6.54 × 10^−10^	Hazra et al. [29]
			Replication: *n* = 1056 Caucasian women	63 (Mean)		Effect: G allele Other: A allele *β* = − 0.08 pg/ml SE = 0.02	1.13 × 10^−6^	
			Combined meta-analysis: *n* = 2714			Effect: G allele Other: A allele *β* = − 0.08 pg/ml SE = 0.01	3.52 × 10^−15^	
19q13.33	Fucosyl transferase 2 gene (*FUT2*)	rs838133	Adults: *n* = 724 Indian	38 ± 11	*T* = 0.205^#^	Effect allele: A *β* = 0.05 pmol/l	> 0.05	Nongmaithem et al. [22]
			PMNS children^*^: *n* = 690 Indian	11 ± 1		Effect allele: A *β* = 0.27 pmol/l	2.00 × 10^−4^	
			PS children^†^: *n* = 534 Indian	5 ± 0		Effect allele: A *β* = 0.06 pmol/l	> 0.05	
19q13.33	Fucosyl transferase 2 gene (*FUT2*)	rs1047781	Initial sample: *n* = 1999 Chinese Han men	38 ± 11	*T* = 0.459	Effect: T allele Other: A allele *β* = not available	4.63 × 10^−17^	Lin et al. [19]
			Replication sample: *n* = 1496 Chinese men	37 ± 11		Effect: T allele Other: A allele *β* = not available	6.79 × 10^−22^	
			Combined total: *n* = 3495			Effect: T allele Other: A allele *β* = 70.21 pg/ml SE = 5.53	3.62 × 10^−36^	
19p13.3	Fucosyltransferase 6 (*FUT6*)	rs708686	Adults: *n* = 724 Indian	38 ± 11	*T* = 0.335^#^	Effect: T allele *β* = 0.13 pmol/l	1.0 × 10^−2^	Nongmaithem et al. [22]
			PMNS children^*^: *n* = 690 Indian	11 ± 1		Effect: T allele *β* = 0.22 pmol/l	2.20 × 10^−4^	
			PS children^†^: *n* = 534 Indian	5 ± 0		Effect: T allele *β* = 0.23 pmol/l	2.70 × 10^−4^	
19p13.3	Fucosyltransferase 6 (*FUT6*)	rs708686	*n* = 25,960 Icelandic	63 ± 24	*T* = 0.301^‡‡^	Effect: T allele Other: C allele *β* = 0.05 pmol/l	2.90 × 10^−9^	Grarup et al. [12]
19p13.3	Fucosyltransferase 6 / Fucosyltransferase 3 (*FUT6*/*FUT3*)	rs3760775	Parents of PMNS cohort^*^: *n* = 1001 Indian	36 ± 5	A = 0.188^#^	Effect allele: A *β* = 0.24 pmol/l	6.00 × 10^−6^	Nongmaithem et al. [22]
			Adults: n = 724 Indian	38 ± 11		Effect allele: A *β* = 0.24 pmol/l	9.90 × 10^−5^	
			PMNS children^*^: *n* = 690 Indian	11 ± 1		Effect allele: A *β* = 0.31 pmol/l	2.90 × 10^−6^	
			PS children^†^: *n* = 534 Indian	5 ± 0		Effect allele: A *β* = 0.24 pmol/l	2.10 × 10^−4^	
19p13.3	Fucosyltransferase 6 (*FUT6*)	rs3760776	Parents of PMNS cohort^*^: *n* = 1001 Indian	36 ± 5	*T* = 0.161^#^	Effect allele: T *β* = 0.10 pmol/l	> 0.05	Nongmaithem et al. [22]
			Adults: *n* = 724 Indian	38 ± 11		Effect allele: T *β* = 0.23 pmol/l	4.40 × 10^−4^	
			PMNS children^*^: *n* = 690 Indian	11 ± 1		Effect allele: T *β* = 0.30 pmol/l	3.30 × 10^−6^	
			PS children^†^: *n* = 534 Indian	5 ± 0		Effect allele: T *β* = 0.18 pmol/l	6.50 × 10^−3^	
19p13.3	Fucosyltransferase 6 (*FUT6*)	rs3760776	*n* = 25,960 Icelandic	63 ± 24	A = 0.071	Effect: A allele Other: G allele *β* = 0.07 pmol/l	4.40 × 10^−6^	Grarup et al. [12]
19p13.3	Fucosyltransferase 6 (*FUT6*)	rs3760776	Initial sample: *n* = 1999 Chinese Han men	38 ± 11	A = 0.212	Effect: A allele Other: G allele *β* = not available	4.23 × 10^−10^	Lin et al. [19]
			Replication sample: *n* = 1496 Chinese men	37 ± 11		Effect: A allele Other: G allele *β* = not available	1.98 × 10^−4^	
			Combined total: *n* = 3495			Effect: A allele Other: G allele *β* = 49.78 pg/ml SE = 6.82	3.68 × 10^−13^	
19p13.3	Fucosyltransferase 6 (*FUT6*)	rs7788053	Icelandic sample: *n* = 37,283	63 ± 24	A = 0.254	Effect: A allele Other: G allele *β* = 0.05 pmol/l	2.10 × 10^−7^	Grarup et al. [12]
			Danish Inter99 population: *n* = 5481	46 ± 8		Effect: A allele Other: G allele *β* = 0.05 pmol/l	> 0.05	
			Danish Health 2006: *n* = 2812	49 ± 13		Effect: A allele Other: G allele *β* = 0.07 pmol/l	7.20 × 10^−4^	
			Combined total: *n* = 45,575			Effect: A allele Other: G allele *β* = not available	1.70 × 10^−10^	
19p13.3	Fucosyltransferase 6 (*FUT6*)	rs78060698	Parents of PMNS cohort^*^: *n* = 1001 Indian	36 ± 5	A = 0.130^††^	Effect allele: A *β* = 0.21 pmol/l	2.90 × 10^−4^	Nongmaithem et al. [22]
			adults: *n* = 724 Indian	38 ± 11		Effect allele: A *β* = 0.20 pmol/l	3.70 × 10^−3^	
			PMNS children^*^: *n* = 690 Indian	11 ± 1		Effect allele: A *β* = 0.27 pmol/l	1.20 × 10^−4^	
			PS children^†^: *n* = 534 Indian	5 ± 0		Effect allele: A *β* = 0.19 pmol/l	8.20 × 10^−3^	
21q22.3	Cystathionine beta synthase (*CBS*)	rs2124459	*n* = 3114 Canadian (85% Causasian, 15% non-Caucasian)	20–79 (range)	C = 0.180	Effect: T allele Other: C allele Vitamin B-12 below adequate (< 220 pmol/l): OR 0.82 (95% CI 0.73, 0.93) pmol/l	2.00 × 10^−3^	Zinck et al. [18]
22q12.2	Transcobalamin 2 (*TCN2*)	rs757874	*n* = 3114 Canadian (85% Causasian, 15% non-Caucasian)	20–79 (range)	*T* = 0.080	Effect: G allele Other: T allele Vitamin B-12 below adequate (< 220 pmol/l): OR 1.42 (95% CI 1.11, 1.72) pmol/l	3.30 × 10^−4^	Zinck et al. [18]
22q12.2	Transcobalamin 2 (*TCN2*)	rs1131603	Adults: *n* = 724 Indian	38 ± 11	C = 0.023^#^	Effect: C allele *β* = 0.43 pmol/l	4.00 × 10^−2^	Nongmaithem et al. [22]
			PMNS children^*^: *n* = 690 Indian	11 ± 1		Effect: C allele *β* = 0.05 pmol/l	> 0.05	
			PS children^†^: *n* = 534 Indian	5 ± 0		Effect: C allele *β* = 0.44 pmol/l	5.00 × 10^−2^	
22q12.2	Transcobalamin 2 (*TCN2*)	rs1131603	Icelandic sample: *n* = 37,283	63 ± 24	C = 0.055	Effect: C allele Other: T allele *β* = 0.19 pmol/l	4.30 × 10^−28^	Grarup et al. [12]
			Danish Inter99 population: *n* = 5481	46 ± 8		Effect: C allele Other: T allele *β* = 0.33 pmol/l	1.80 × 10^−9^	
			Danish Health 2006: *n* = 2812	49 ± 13		Effect: C allele Other: T allele *β* = 0.33 pmol/l	5.30 × 10^−17^	
			Combined total: *n* = 45,575			Effect: C allele Other: T allele *β* = not available	4.90 × 10^−49^	
22q12.2	Transcobalamin 2 (*TCN2*)	rs5753231	Icelandic sample: *n* = 25,960	63 ± 24	*T* = 0.210	Effect: C allele Other: T allele *β* = 0.06 pmol/l	7.50 × 10^−10^	Grarup et al. [12]

All studies have a cross-sectional study design

*SNP* single-nucleotide polymorphism

*Pune Maternal Nutrition Study (PMNS)

^†^Parthenon Study (PS)

^‡^Nurses’ Health Study (NHS) NCI-Cancer Genetic Markers of Suceptibility (CGEMS) project

^§^Framingham-SNP-Health Association Resource (SHARe)

^¶^Baltimore Longitudinal Study of Aging (BLSA)

^#^Data refers to the HapMap-GIH population, with data collected from Gujarati Indians from Houston, Texas

^**^Data refers to European populations collected from: Utah Residents (CEPH) with Northern and Western European Ancestry, Toscani in Italia, Finnish in Finland, British in England and Scotland and Iberian Population in Spain

^††^Data refers to South Asian populations collected from: Gujarati Indian from Houston, Texas, Punjabi from Lahore, Pakistan, Bengali from Bangladesh, Sri Lankan Tamil from the UK and Indian Telugu from the UK

^‡^Data refers to the HapMap-CEU population, with data collected from Utah Residents (CEPH) with Northern and Western European Ancestry

**Table 2:** Candidate gene association studies examining the association of SNPs with vitamin B12 concentrations. Candidate gene association studies testing the association between SNPs and vitamin B12 concentrations. The chromosome location, gene name, reference SNP cluster ID, sample size and ethnicity, study design, mean age, observed frequency of the minor allele in the population, effect size and *P* value are shown in the table. The order of SNPs reflects the order of the chromosome location

Chromosome location	Gene name (gene symbol)	Reference SNP cluster ID	Sample size and ethnicity	Study design	Age (years)	Minor allele frequency	Effect size	*P* value	References
1p34.1	Methylmalonic aciduria and homocystinuria type C protein (*MMACHC*)	rs10789465	*n* = 262 Caucasian women of North European descent	Cross-sectional (Twin Study)	48 ± 13	C = 0.469^†^	Not available	1.00 × 10^−3^	Andrew et al. [13]
1p36.3	Methylenetetrahydrofolate Reductase (*MTHFR*)	rs1801131	*n* = 988 French women	Cross-sectional	40–65 (range)	C = 0.290	Not available	> 0.05	De Batlle et al. [79]
1p36.3	Methylenetetrahydrofolate reductase (*MTHFR*)	Rs1801131	*n* = 6784 Danish	Cross-sectional	30–60 (range)	C = 0.340	Not available	> 0.05	Thuesen et al. [57]
1p36.3	Methylenetetrahydrofolate Reductase (*MTHFR*)	Rs1801131	*n* = 220 Brazilian	Cross-sectional	1–8 (range)	C = 0.240	Not available	> 0.05	Aléssio et al. [78]
1p36.3	Methylenetetrahydrofolate Reductase (*MTHFR*)	rs1801133	*n* = 988 French women	Cross-sectional	40–65 (range)	*T* = 0.360	Not available	> 0.05	De Batlle et al. [79]
1p36.3	Methylenetetrahydrofolate Reductase (*MTHFR*)	rs1801133	*n* = 731 English (White Caucasian)	Cross-sectional	85	*T* = 0.330	*β* = 5.00 × 10^−5^ pmol/l^‡^	> 0.05	Mendonca et al. [28]
1p36.3	Methylenetetrahydrofolate Reductase (*MTHFR*)	rs1801133	Elderly individuals: *n* = 262 Brazilian	Cross-sectional	60–91 (range)	*T* = 0.370	Not available	> 0.05	Barnabe et al. [77]
			Children: *n* = 106 Brazilian		0.5–6 (range)	*T* = 0.290	Not available	> 0.05	
1p36.3	Methylenetetrahydrofolate Reductase (*MTHFR*)	rs1801133	*n* = 6784 Danish	Cross-sectional	30–60 (range)	T = 0.290	Effect allele: Not available Other allele: not available Low serum vitamin B12: OR 1·78 (95% CI 1.25, 2.54) pmol/l	3.00 × 10^−3^	Thuesen et al. [57]
1p36.3	Methylenetetrahydrofolate Reductase (*MTHFR*)	rs1801133	*n* = 153 Spanish	Cross-sectional	13–19 (range)	*T* = 0.380	Not available	> 0.05	Al-Tahan et al. [81]
1p36.3	Methylenetetrahydrofolate Reductase (*MTHFR*)	rs1801133	*n* = 10,601 Norwegian	Cross-sectional	56	*T* = 0.280	Not available	> 0.05	Hustad et al. [80]
1p36.3	Methylenetetrahydrofolate Reductase (*MTHFR*)	rs1801133	*n* = 220 Brazilian	Cross-sectional	1–8 (range)	*T* = 0.320	Not available	> 0.05	Aléssio et al. [78]
1q43	5-Methyltetrahydrofolate-Homocysteine methyltransferase (MTR)	rs1805087	*n* = 731 English (White Caucasian)	Cross-sectional	85	G = 0.180	*β* = 4.00 × 10^−3^ pmol/l^‡^	> 0.05	Mendonca et al. [28]
1q43	5-Methyltetrahydrofolate-Homocysteine methyltransferase (MTR)	rs1805087	*n* = 262 Caucasian women of North European descent	Cross-sectional (Twin Study)	48 ± 13	G = 0.161^†^	Not available	> 0.05	Andrew et al. [13]
1q43	5-Methyltetrahydrofolate-Homocysteine methyltransferase (MTR)	rs1805087	*n* = 6784 Danish	Cross-sectional	30–60 (range)	G = 0.200	Not available	> 0.05	Thuesen et al. [57]
1q43	5-Methyltetrahydrofolate-Homocysteine methyltransferase (MTR)	rs2275568	*n* = 262 Caucasian women of North European descent	Cross-sectional (Twin Study)	48 ± 13	A = 0.460^†^	Not available	> 0.05	Andrew et al. [13]
1q43	5-Methyltetrahydrofolate-Homocysteine methyltransferase (MTR)	rs2789352	*n* = 262 Caucasian women of North European descent	Cross-sectional (Twin Study)	48 ± 13	*T* = 0.381^†^	Not available	> 0.05	Andrew et al. [13]
1q43	5-Methyltetrahydrofolate-Homocysteine methyltransferase (MTR)	rs3768142	*n* = 262 Caucasian women of North European descent	Cross-sectional (Twin Study)	48 ± 13	G = 0.384^†^	Not available	> 0.05	Andrew et al. [13]
1q43	5-Methyltetrahydrofolate-homocysteine methyltransferase (MTR)	rs10733118	*n* = 262 Caucasian women of North European descent	Cross-sectional (Twin Study)	48 ± 13	T = 0.381^†^	Not available	> 0.05	Andrew et al. [13]
1q43	5-Methyltetrahydrofolate-Homocysteine methyltransferase (MTR)	rs10925257	*n* = 262 Caucasian women of North European descent	Cross-sectional (Twin Study)	48 ± 13	G = 0.155^†^	Not available	> 0.05	Andrew et al. [13]
1q43	5-Methyltetrahydrofolate-homocysteine methyltransferase (MTR)	rs11800413	*n* = 262 Caucasian women of North European descent	Cross-sectional (Twin Study)	48 ± 13	G = 0.431^†^	Not available	> 0.05	Andrew et al. [13]
1q43	5-Methyltetrahydrofolate-homocysteine methyltransferase (MTR)	rs12060264	*n* = 262 Caucasian women of North European descent	Cross-sectional (Twin Study)	48 ± 13	A = 0.438^†^	Not available	> 0.05	Andrew et al. [13]
2q23.2	Methylmalonic aciduria and homocystinuria,CblD type (*MMADHC*)	rs7580915	*n* = 262 Caucasian women of North European descent	Cross-sectional (Twin Study)	48 ± 13	G = 0.228^†^	Not available	> 0.05	Andrew et al. [13]
4p14	Replication factor C subunit 1 (*RFC1*)	rs1051266	Elderly individuals: *n* = 262 Brazilian	Cross-sectional	60–91 (range)	A = 0.430	Not available	> 0.05	Barnabe et al. [77]
			Children: *n* = 106 Brazilian		1–6 (range)	A/G = 0.500	Not available	> 0.05	
4q31.21	Methylmalonic aciduria (cobalamin deficiency) cblA type (*MMAA*)	rs4835011	*n* = 262 Caucasian women of North European descent	Cross-sectional (Twin Study)	48 ± 13	G = 0.080^†^	Not available	> 0.05	Andrew et al. [13]
4q31.21	Methylmalonic aciduria (cobalamin deficiency) cblA type (*MMAA*)	rs4835012	*n* = 262 Caucasian women of North European descent	Cross-sectional (Twin Study)	48 ± 13	G = 0.178^†^	Not available	3.00 × 10^−2^	Andrew et al. [13]
4q31.21	Methylmalonic aciduria (cobalamin deficiency) cblA type (*MMAA*)	rs4835014	*n* = 262 Caucasian women of North European descent	Cross-sectional (Twin Study)	48 ± 13	*T* = 0.031^†^	Not available	> 0.05	Andrew et al. [13]
4q31.21	Methylmalonic aciduria (cobalamin deficiency) cblA type (*MMAA*)	rs11728906	*n* = 262 Caucasian women of North European descent	Cross-sectional (Twin Study)	48 ± 13	G = 0.235^†^	Not available	> 0.05	Andrew et al. [13]
5q14.1	Betaine-homocysteine S-methyltransferase (*BHMT*)	rs3733890	*n* = 6784 Danish	Cross-sectional	30–60 (range)	A = 0.290	Not available	> 0.05	Thuesen et al. [57]
5p15.31	Methionine synthase reductase (*MTRR*)	rs10380	*n* = 262 Caucasian women of North European descent	Cross-sectional (Twin Study)	48 ± 13	*T* = 0.156^†^	Not available	> 0.05	Andrew et al. [13]
5p15.31	Methionine synthase reductase (*MTRR*)	rs162031	*n* = 262 Caucasian women of North European descent	Cross-sectional (Twin Study)	48 ± 13	*T* = 0.205^†^	Not available	> 0.05	Andrew et al. [13]
5p15.31	Methionine synthase reductase (*MTRR*)	rs162036	*n* = 262 Caucasian women of North European descent	Cross-sectional (Twin Study)	48 ± 13	G = 0.186^†^	Not available	4.00 × 10^−2^	Andrew et al. [13]
5p15.31	Methionine synthase reductase (*MTRR*)	rs162040	*n* = 262 Caucasian women of North European descent	Cross-sectional (Twin Study)	48 ± 13	C = 0.124^†^	Not available	> 0.05	Andrew et al. [13]
5p15.31	Methionine synthase reductase (*MTRR*)	rs162048	*n* = 262 Caucasian women of North European descent	Cross-sectional (Twin Study)	48 ± 13	G = 0.164^†^	Not available	5.00 × 10^−2^	Andrew et al. [13]
5p15.31	Methionine synthase reductase (*MTRR*)	rs326120	*n* = 262 Caucasian women of North European descent	Cross-sectional (Twin Study)	48 ± 13	G = 0.155^†^	Not available	> 0.05	Andrew et al. [13]
5p15.31	Methionine synthase reductase (*MTRR*)	rs1532268	*n* = 262 Caucasian women of North European descent	Cross-sectional (Twin Study)	48 ± 13	A = 0.308^†^	Not available	1.00 × 10^−2^	Andrew et al. [13]
5p15.31	Methionine synthase reductase (*MTRR*)	rs1801394	*n* = 6784 Danish	Cross-sectional	30–60 (range)	A = 0.430	Not available	> 0.05	Thuesen et al. [57]
5p15.31	Methionine synthase reductase (*MTRR*)	rs1801394	*n* = 220 Brazilian	Cross-sectional	1–8 (range)	A = 0.490	Not available	> 0.05	Aléssio et al. [78]
5p15.31	Methionine synthase reductase (*MTRR*)	rs2966952	*n* = 262 Caucasian women of North European descent	Cross-sectional (Twin Study)	48 ± 13	*T* = 0.167^†^	Not available	> 0.05	Andrew et al. [13]
5p15.31	Methionine synthase reductase (*MTRR*)	rs3776455	*n* = 262 Caucasian women of North European descent	Cross-sectional (Twin Study)	48 ± 13	G = 0.389^†^	Not available	2.00 × 10^−3^	Andrew et al. [13]
5p15.31	Methionine synthase reductase (*MTRR*)	rs6555501	*n* = 262 Caucasian women of North European descent	Cross-sectional (Twin Study)	48 ± 13	C = 0.473^†^	Not available	> 0.05	Andrew et al. [13]
6p12.3	Methylmalonyl-CoA mutase (*MUT*)	rs6458687	*n* = 262 Caucasian women of North European descent	Cross-sectional (Twin Study)	48 ± 13	*T* = 0.372^†^	Not available	> 0.05	Andrew et al. [13]
6p12.3	Methylmalonyl-CoA mutase (*MUT*)	rs6458690	*n* = 262 Caucasian women of North European descent	Cross-sectional (Twin Study)	48 ± 13	G = 0.363^†^	Not available	2.00 × 10^− 4^	Andrew et al. [13]
6p12.3	Methylmalonyl-CoA mutase (*MUT*)	rs9381784	*n* = 262 Caucasian women of North European descent	Cross-sectional (Twin Study)	48 ± 13	*T* = 0.363^†^	Not available	3.00 × 10^−2^	Andrew et al. [13]
6q13	LMBR1 domain containing 1 (*LMBRD1*)	rs991974	*n* = 262 Caucasian women of North European descent	Cross-sectional (Twin Study)	48 ± 13	*T* = 0.044^†^	Not available	> 0.05	Andrew et al. [13]
6q13	LMBR1 domain containing 1 (*LMBRD1*)	rs1457498	n = 262 Caucasian women of North European descent	Cross-sectional (Twin Study)	48 ± 13	*T* = 0.084^†^	Not available	> 0.05	Andrew et al. [13]
6q13	LMBR1 domain containing 1 (*LMBRD1*)	rs3778241	*n* = 262 Caucasian women of North European descent	Cross-sectional (Twin Study)	48 ± 13	*T* = 0.398^†^	Not available	> 0.05	Andrew et al. [13]
6q13	LMBR1 domain containing 1 (*LMBRD1*)	rs3799105	*n* = 262 Caucasian women of North European descent	Cross-sectional (Twin Study)	48 ± 13	C = 0.384^†^	Not available	> 0.05	Andrew et al. [13]
6q13	LMBR1 domain containing 1 (*LMBRD1*)	rs6455338	*n* = 262 Caucasian women of North European descent	Cross-sectional (Twin Study)	48 ± 13	C = 0.387^†^	Not available	> 0.05	Andrew et al. [13]
6q13	LMBR1 domain containing 1 (*LMBRD1*)	rs9294851	*n* = 262 Caucasian women of North European descent	Cross-sectional (Twin Study)	48 ± 13	*T* = 0.384^†^	Not available	> 0.05	Andrew et al. [13]
11q12.1	Transcobalamin 1 (*TCN1*)	rs526934	*n* = 731 English (White Caucasian)	Cross-sectional	85	G = 0.270	*β* = 4.00 × 10^−3^ pmol/l^‡^	> 0.05	Mendonca et al. [28]
12q24.11	Methylmalonic aciduria (cobalamin deficiency) cblB type (*MMAB*)	rs2287182	*n* = 262 Caucasian women of North European descent	Cross-sectional (Twin Study)	48 ± 13	A = 0.128^†^	Not available	> 0.05	Andrew et al. [13]
12q24.11	Methylmalonic aciduria (cobalamin deficiency) cblB type (*MMAB*)	rs3759387	*n* = 262 Caucasian women of North European descent	Cross-sectional (Twin Study)	48 ± 13	A = 0.235^†^	Not available	> 0.05	Andrew et al. [13]
12q24.11	Methylmalonic aciduria (cobalamin deficiency) cblB type (*MMAB*)	rs7134594	*n* = 262 Caucasian women of North European descent	Cross-sectional (Twin Study)	48 ± 13	C = 0.487^†^	Not available	> 0.05	Andrew et al. [13]
12q24.11	Methylmalonic aciduria (cobalamin deficiency) cblB type (*MMAB*)	rs7957619	*n* = 262 Caucasian women of North European descent	Cross-sectional (Twin Study)	48 ± 13	A = 0.110 ^†^	Not available	> 0.05	Andrew et al. [13]
12q24.11	Methylmalonic aciduria (cobalamin deficiency) cblB type (*MMAB*)	rs12314392	*n* = 262 Caucasian women of North European descent	Cross-sectional (Twin Study)	48 ± 13	G = 0.433 ^†^	Not available	> 0.05	Andrew et al. [13]
19p13.2	CD320 molecule (*CD320*)/transcobalamin II receptor (*TcblR*)	rs2336573	*n* = 591 Caucasian women	Cross-sectional	77 ± 7	A = 0.050	Not available	> 0.05	Kurnat-Thoma et al. [59]
			*n* = 198 African American women		75 ± 6	A = 0.330	Not available	4.0 × 10^−2^	
			*n* = 797 Combined total				Not available	2.0 × 10^−2^	
19q13.33	Fucosyl transferase 2 gene (*FUT2*)	rs492602	*n* = 731 English (White Caucasian)	Cross-sectional	85	A = 0.450	*β* = 0.05 pmol/l^‡^	< 0.001	Mendonca et al. [28]
19q13.33	Fucosyl transferase 2 gene (*FUT2*)	rs602662	Vegetarian: *n* = 553 North Indian	Cross-sectional	50 (41–59) Median (interquartile range)	A = 0.310	Effect: A allele Other: G allele *β* = 0.12 pmol/l	5.0 × 10^−3^	Tanwar et al. [27]
			Non-vegetarian: *n* = 593 North Indian	Cross-sectional	47 (37–55) Median (interquartile range)		Effect: A allele Other: G allele *β* = 0.12 pmol/l	4.0 × 10^−3^	
			Combined total: *n* = 1146 North Indian	Cross-sectional	49 (40–57) Median (interquartile range)		Effect: A allele Other: G allele *β* = 0.12 pmol/l	4.0 × 10^−5^	
22q12.2	Transcobalamin 2 (*TCN2*)	rs1801198	NORCAP cohort^*^: *n* = 2411 Norwegian Serum holoTC could be analysed in only 2379 individuals	Cross-sectional	50–64 (range)	G = 0.440	Effect: C allele Other: G allele Total holo-TC: *β* = 0.02 pmol/l^‡^	> 0.05^‡^	Riedel et al. [55]
							Effect: C allele Other: G allele Plasma Cbl: *β* = 0.03 pmol/l^‡^	> 0.05^‡^	
22q12.2	Transcobalamin 2 (*TCN2*)	rs1801198	*n* = 122 Portuguese	Cross-sectional	46 ± 13	G = 0.480	Not available	Vitamin B12: > 0.05 Holo-TC: < 0.05	Castro et al. [52]
22q12.2	Transcobalamin 2 (*TCN2*)	rs1801198	*n* = 554 Participants of Latino ancestry residing in USA	Cross-sectional	69 ± 6	G = 0.350	Not available	Vitamin B12: > 0.05 Total holo TC: > 0.05	Garrod et al. [56]
22q12.2	Transcobalamin 2 (*TCN2*)	rs1801198	*n* = 613 Northern Irish Men (Caucasian)	Cross-sectional	30–49 (range)	G = 0.450	Not available	1.00 × 10^−2^	Stanislawska-Sachadyn et al. [54]
22q12.2	Transcobalamin 2 (*TCN2*)	rs1801198	*n* = 6784 Danish	Cross-sectional	30–60 (range)	G = 0.440	Not available	> 0.05	Thuesen et al. [57]
22q12.2	Transcobalamin 2 (*TCN2*)	rs1801198	*n* = 207 Brazillian	Cross-sectional	1–8 (range)	G = 0.360	Not available	> 0.05	Alessio et al. [58]
22q12.2	Transcobalamin 2 (*TCN2*)	rs4820888	*n* = 591 Caucasian women	Cross-sectional	77 ± 7	G = 0.430	Not available	> 0.05	Kurnat-Thoma et al. [59]
			*n* = 198 African American women		75 ± 6	G = 0.450	Not available	> 0.05	
			*n* = 797 Combined total				Not available	2.0 × 10^−2^	
22q12.2	Transcobalamin 2 (*TCN2*)	rs9606756	NORCAP cohort^*^: *n* = 2411 Norwegian Serum holoTC could be analysed in only 2379 individuals	Cross-sectional	50–64 (range)	G = 0.120	Effect: A allele Other: G allele Total holo-TC: *β* = − 0.21 pmol/l^‡^	< 0.001^‡^	Riedel et al. [55]
							Effect: A allele Other: G allele Plasma Cbl: *β* = −0.02 pmol/l^‡^	> 0.05^‡^	
22q12.2	Transcobalamin 2 (*TCN2*)	rs9606756	*n* = 6784 Danish	Cross-sectional	30–60 (range)	G = 0.120	Not available	> 0.05	Thuesen et al. [57]
1p36.3 19q13.33	Methylenetetrahydrofolate Reductase (*MTHFR*) + Fucosyl transferase 2 gene (FUT2)	rs180133 rs180131 rs492602	*n* = 988 Brazillian	Cross-sectional	5 ± 3	rs180133 T = 0.320 rs180131 C = 0.220 rs492602 G = 0.390	*β* for GRS = − 0·11 pmol/l	< 0·001	Cobayashi et al. [105]

All studies have a cross-sectional design

SNP single-nucleotide polymorphism

^*^NORwegian Colorectal CAncer Prevention (NORCCAP) cohort

^†^Data refers to HapMap European population, with data collected from Utah Residents (CEPH) with Northern and Western European Ancestry

^‡^The specific data available is not published elsewhere and was obtained by contacting the corresponding authors

**Table 3:** A summary of the most frequently studied genes associated with vitamin B12 concentrations. The gene name, gene location and function of the most frequently studied genes associated with vitamin B12 concentrations are summarized in this table

Vitamin B12-related proteins	Gene name	Location	Function
Co-factors or regulators of co-factors essential for the transport of vitamin B12	Methylmalonic aciduria and homocystinuria, cblC type (*MMACHC)*	1p34.1	The *MMACHC* gene encodes a chaperone protein MMAACHC (cblC protein) which binds to vitamin B12 in the cytoplasm and appears to catalyze the reductive decyanation of cyanocobalamin into cob(II)alamin [11].
	Transcobalamin 1 (*TCN1*)	11q12.1	It encodes a glycoprotein called Transcobalamin 1, also known as haptocorrin (HC), which binds to vitamin B12. It shields dietary vitamin B12 from the acidic environment of the stomach [12,18–22,108].
	Fucosyltransferase 2 (*FUT2*)	19q13.33	It encodes the enzyme fucosyltransferase 2 (FUT2), which is involved in the synthesis of antigens of the Lewis blood group [5]. These antigens mediate the attachment of gastric pathogens to the gastric mucosa, which can affect the absorption of vitamin B12 [109].
	Fucosyltransferase 6 (*FUT6*)	19p13.3	It encodes the enzyme fucosyltransferase 6 (FUT6), which is involved in forming Lewis associated antigens. These antigens attach gastric pathogens to the gastric mucosa. It has been shown that these gastric pathogens can reduce the absorption of vitamin B12 in the gut [43,44].
	Transcobalamin 2 (*TCN2*)	22q12.2	It encodes a transport protein called transcobalamin 2 (TC), which binds to vitamin B12 within the enterocyte. The TC-B12 complex enters the portal circulation [59] and makes vitamin B12 available for cellular uptake in target tissues [49,110].
Membrane transporters that actively facilitates membrane crossing	Cubilin (*CUBN*)	10p13	It encodes the intestinal receptor Cubilin, which is expressed in the renal proximal tubule and intestinal mucosa [20,60,61]. Cubilin recognizes the vitaminB12-intrinsic factor complex, and binds to another protein called Amnionless to facilitate the entry of vitamin B12 into the intestinal cells [67].
	ATP binding cassette subfamily D member 4 (*ABCD4*)	14q24.3	*ABCD4* codes for an ABC transporter. It has been postulated that ABCD4 is involved in intracellular cobalamin processing [69], and is involved in transporting vitamin B12 from lysosomes to the cytosol. In the cytosol, vitamin B12 can be converted into methylcobalamin (MeCbl) and adenosylcobalamin (AdoCbl) [70].
	CD320 molecule (*CD320*)	19p13.2	It encodes the membrane receptor transcobalamin receptor (TCblR), which binds to the transcobalamin-vitamin B12 complex, and mediates the uptake of vitamin B12 into cells [72].
Proteins involved in the catalysis of enzymatic reactions in the one carbon cycle	Methylenetetrahydrofolate reductase (*MTHFR*)	1p36	*MTHFR* codes for a critical enzyme involved in homocysteine remethylation. MTHFR catalyzes the reduction of 5,10-methylenetetrahydrofolate to 5-methyltetrahydrofolate in an irreversible reaction [74].
	Methionine synthase reductase (*MTRR*)	5p15.31	This gene is responsible for maintaining adequate levels of activated vitamin B12 (methylcob(III)alamin), which maintains the enzyme methionine synthase in its active state [83].
Proteins involved in cell cycle regulation	Membrane-spanning 4-domains A3 (*MS4A3*)	11q12.1	*MS4A3* encodes a protein involved as a hematopoietic cell cycle regulator [85]. *MS4A3* gene may have a role in the cell-cycle regulation in the GI tract, thus affecting the renewal of intestinal and gastric epithelial cells, and absorption of vitamin B12 [86].
Mitochondrial protein	Methylmalonic aciduria (cobalamin deficiency) cb1A type (*MMAA*)	4q31	*MMAA* encodes a protein that may be involved in the translocation of vitamin B12 into the mitochondria [88]. In addition, MMAA could play an important role in the protection and reactivation of Methylmalonyl-coA mutase (MCM) in vitro [90].
	Methylmalonyl-CoA mutase (*MUT*)	6p12.3	It encodes a Mitochondrial enzyme methylmalonyl-CoA mutase (MUT), which catalyzes the isomerization of methylmalonyl-CoA to succinyl-CoA. This isomerization requires vitamin B12 as a cofactor in the form of 5-prime-deoxyadenosylcobalamin (AdoCbl) [91].
	Citrate lyase beta like (*CLYBL*)	13q32.3	It encodes a human mitochondrial enzyme, which is co-expressed with other co-enzymes in the mitochondrial B12 pathway [111].

## Co-factors or regulators of co-factors essential for the transport of vitamin B12

### Methylmalonic aciduria and homocystinuria, cblC type (*MMACHC)*

The methylmalonic aciduria and homocystinuria, cblC type (*MMACHC*) gene is located in the chromosome region 1p34.1 [10]. The *MMACHC* gene encodes a chaperone protein MMACHC (cblC protein) which binds to vitamin B12 in the cytoplasm and appears to catalyze the reductive decyanation of cyanocobalamin into cob(II)alamin [11].

Among the common variations, SNP rs12272669 has been associated with vitamin B12 status, where ‘A’ allele carriers had higher vitamin B12 concentrations compared with ‘G’ allele carriers (*P* = 3.00 × 10^−9^, *β* = 0.51 pmol/l) in 37,283 Icelandic individuals [12]. Furthermore, SNP rs10789465 was associated with vitamin B12 concentrations (*P* = 1.00 × 10^−3^) in a candidate gene association study comprising 262 Caucasian women of North European descent [13]. Currently, it is unknown how these variants affect the regulation of the *MMACHC* gene.

### Transcobalamin 1 (*TCN1*)

The transcobalamin 1 (*TCN1*) gene is located on chromosome 11 and codes for the vitamin B12 binding protein, transcobalamin I (TCI; also called haptocorrin (HC) or R binder) [14–16]. TCI is involved in facilitating the entry of vitamin B12 into the cells, via receptor-mediated endocytosis [17]. Six studies have reported associations between variants within the *TCN1* gene and circulating vitamin B12 concentrations [12, 18–22].

Nongmaithem et al. [22] investigated the association between several nucleotide variations within the *TCN1* gene and vitamin B12 levels in a GWA study comprising 534 healthy children from Mysore, India. Carriers of the ‘G’ allele of the rs526934 variant were found to have lower circulating vitamin B12 concentrations (β = − 0.16 pmol/l, *P* = 0.02) compared to ‘A’ allele carriers [22]. This finding was in accordance with the studies conducted in Chinese, Icelandic, Italian and individuals residing in the US (predominantly non-Hispanic white) [12, 19–21]. Furthermore, additional variants of the *TCN1* gene (rs34528912 and rs34324219) were observed to be associated with vitamin B12 status (*P* < 0.05) in individuals of Icelandic, Indian and Danish backgrounds [12, 22].

Although no functional data are available to confirm the functional effect of these SNPs on vitamin B12 concentrations, the results from these studies suggest that the SNPs may have important physiological consequences for the role of the TCN1 protein in relation to vitamin B12 levels.

### Fucosyltransferase 2 (*FUT2*)

The fucosyltransferase 2 *(FUT2* gene*),* also known as the Se gene (secretor) is located on chromosome 19. The *FUT2* gene codes for a secretor enzyme α(1,2) fucosyltransferase which fucosylates oligosaccharides producing H type 1 and 2 antigens. H antigens are precursors of ABO and Lewis b histo-blood group antigens that are expressed on mucosal surfaces [5]. Recent studies have shown suggestive associations between variants of *FUT2* with diabetes and body mass index [23–26].

For the *FUT2* gene, seven SNPs including rs281379, rs492602, rs516316, rs601338, rs602662, rs838133 and rs1047781 were previously reported to be associated with vitamin B12 levels [12, 18–22, 27–29]. To identify loci associated with plasma vitamin B12, a meta-analysis of three genome-wide association scans (*n* = 4763) was carried out in a Caucasian population residing in the USA [20]. The SNP rs601338, also known as 428 G/A nonsecretor variant allele (W143X variant), was significantly associated with plasma vitamin B12 levels (*P* = 6.92 × 10^−15^), with the allele ‘A’ being positively associated with plasma vitamin B12 levels (*β =* 0.06 pg/ml) [20]. This finding was further confirmed in another study looking at 37,283 Icelandic adults (*P* = 2.40 × 10^−95^, *β =* 0.162 pmol/l) [12], as well as in two Indian populations of children (*β =* 0.18–0.25 pmol/l) [22]. Notably, the minor allele frequency (MAF) of rs601338 varies widely between ethnicities, contributing to genetic heteroegeneity in *FUT2*-B12 associations. In previous reports by Grarup et al. [12] and Hazra et al. [29], the frequency of the minor allele ‘G’ for the associated SNP (rs601338) was between 38.4 and 49.0%, for Icelandic and Caucasian populations from the USA, respectively. In contrast, the allele ‘A’ was found to be the minor allele in the Indian population (MAF = 23.0%) [22]. The presence of the ‘A’ allele is associated with higher vitamin B12 concentrations, compared to ‘G’ allele carriers. This indicates that in the Indian population, a greater number of individuals carry the ‘G’ allele and hence could partly explain why Indians are expected to have a lower vitamin B12 status [27]. The *FUT2* rs601338 variant is less common in East Asians than Europeans [MAF = 3.5%; HapMap HCB (Han Chinese in Beijing, China) and MAF = 1.2%; HapMap JPT (Japanese in Tokyo, Japan)] and may explain why the locus has not been identified in Chinese individuals in previous studies [19]. Another common non-synomynous SNP rs1047781 (A385T) has been shown to be a potential functional variant associated with vitamin B12 status and a major FUT2 secretor defining SNP in East Asians, and has also been reported to reduce the expression of Fucosyltransferases [30, 31]. Lin et al. found that the ‘T’ allele of the SNP rs1047781 was significantly associated with higher vitamin B12 concentrations in 3495 Chinese men (*P* = 3.62 × 10^−36^, *β* = 70.21 pg/ml) [19]. This genetic marker is present only in East-Asians; hence, it could not be replicated in a study conducted in Icelandic individuals [12].

To date, three studies have shown an association between the SNP rs492602 and vitamin B12 concentrations [18, 20, 29]. The SNP rs492602 is in complete linkage disequilibrium (LD) with *FUT2* W143X (rs601338) (*r*^2^ = 1), as shown in the Nurses Health Study [29]. Hazra et al. [20] found that the ‘A’ allele of the SNP rs492602 variant was associated with lower vitamin B12 concentrations (*β* = − 0.06 pg/ml, *P* = 1.30 × 10^−14^) among 4763 Caucasians from the USA, this finding was similarly observed in a GWA study (2696 women) by the same authors (*β* = − 0.09 pg/ml, *P* = 5.36 × 10^−17^) [29]. In a subsequent study in 3114 Canadian adults, the ‘G’ allele was shown to be associated with a lower risk (*P* = 2.0 × 10^−4^, odds ratio 0.60, 95% CI 0.54–0.70) of vitamin B12 deficiency (< 148 pmol/l) [18].

Finally, the most commonly studied variant of the *FUT2* gene is the SNP rs602662. This SNP was also reported to be in LD with the SNPs rs601338 (*r*^2^ = 0.76) and rs516316 (*r*^2^ = 0.83) in Caucasian populations from the USA and Iceland [12, 29]. Zinck et al. [18] reported that ‘A’ allele carriers of the rs602662 variant were at a lower risk of vitamin B12 deficiency (< 148 pmol/l) (OR 0.61, 95% CI 0.47–0.80, *P* = 3.0 × 10^−4^) in a population of 3114 Canadian adults [18]. Similarly, a higher vitamin B12 status was observed in carriers of the ‘A’ allele in four different studies looking at Caucasians (*β =* 0.04–43.27 pmol/l) [12, 20, 21, 29] and Indians (*β =* 0.10–0.25 pmol/l) [22, 27]. Furthermore, additional variants of the *FUT2* gene were observed to be associated with vitamin B12 levels (*P* < 0.05) in the following SNPs: rs1047781, rs516316, rs838133 and rs281379 [12, 19, 22].

It has been proposed that host genetic variation in the *FUT2* gene may alter the composition of the gut microbiome. Individuals, who are nonsecretors (homozygous for the non-functional *FUT2* phenotype), lack terminal fucose residues on mucin glycans [32, 33]. As a result, the gut microbial community of individuals with FUT2 deficiency may reduce in composition and diversity, as microbes cannot adhere or utilize host-derived glycans [33, 34]. Variations in the *FUT2* gene can potentially alter the susceptibility to *Helicobacter pylori* (*H. pylori)* infection and its related gastric-induced vitamin B12 malabsorption [35–40]. Gastric pathogens, such as *H. pylori*, attach to α1,2-fucosylated glycan’s on epithelial cells, or structures masked by fucosylation with the help of these H antigens in individuals with the secretor status [35–40]. Infections with *H. pylori* in the human intestine have been reported to interfere with the release of intrinsic factor needed for vitamin B12 absorption [40]. Interestingly, a study in Northern Portugal found that the SNP rs602662 ‘A’ allele has been linked to a non-secretor status (null H antigens), and this may decrease the risk of bacterial infection from pathogens, such as *H. pylori*, and explains why subjects who carry ‘A’ allele have a high vitamin B12 status [41]. Alternatively, independent of *H. pylori*-mediated gastritis, individuals who carried *FUT2* secretor variants who were also heterozygous for a *GIF* (a fucosylated glycoprotein needed for vitamin B12 absorption) mutation, had lower vitamin B12 concentrations [42].

### Fucosyltransferase 6 (*FUT6*)

The fucosyltransferase 6 (*FUT6*) gene is located on chromosome 19 and encodes a Golgi stack membrane protein, involved in the formation of Sialyl-Lewis X, an E-selectin ligand [19]. These Lewis associated antigens are associated with *H. pylori* adherence to the gastric and duodenal mucosa [43, 44]. Overgrowth of *H. pylori* has been linked to vitamin B12 deficiency, as gastric bacteria reduces the secretion of IF which is needed to form the vitaminB12-IF complex [19, 40].

In light of the potential physiological link between the *FUT6* gene and vitamin B12 deficiency, three studies investigated the relationship between variants in the *FUT6* gene and vitamin B12 status. Lin et al. first observed [19] that the ‘A’ allele of the rs3760776 variant was associated with higher vitamin B12 levels (*β =* 49.78 pg/ml, *P* = 3.68 × 10^−13^) in a sample of 3495 men of Chinese Han and Chinese descent [19]. Similarly, homozygous ‘A’ allele carriers of Icelandic (*β* = 0.068 pmol/l, *P* = 4.4 × 10^−6^) [12] and Indian (*β =* 0.18–0.30 pmol/l) [22] populations had high serum vitamin B12 concentrations. Interestingly, this gene variant may have the potential to serve as a genetic marker for type 2 diabetes [26].

Furthermore, additional variants of the *FUT6* gene (rs708686 [12, 22], rs78060698 [22], rs3760775 [22] and rs7788053 [12]) were observed to be associated with a higher vitamin B12 status in individuals of the Indian, Icelandic and Danish populations (*P* < 0.05). Bioinformatic analysis has shown that the *FUT3*, *FUT5* and *FUT6* genes form a cluster on chromosome 19p13.3 [45]. Interestingly, the SNPs rs3760775, rs10409772, rs12019136, rs78060698, rs17855739, rs79744308, rs7250982 and rs8111600 from this cluster were in LD with the *FUT6* SNP rs3760775 (*r*^2^ = 0.57–0.84) in South Asian populations. Available data has shown differences in the LD structure between South Asian populations and individuals of East Asian and European origin [22]. The variation of LD patterns across ethnicities could account for the heterogeneity of vitamin B12 concentrations [46].

Nongmaithem et al. [22] noted that alternative allelic states of the SNP rs78060698 variant may influence the binding affinity of HNF4α (a key regulator of FUT6 expression) to the FUT6 protein. FUT6 is responsible for synthesizing α(1,3) fucosylated glycans, which act as a biological interface for the host-microbial interaction [47]. It is plausible that the SNP rs78060698 maintains the structure of glycans, which in turn control intestinal host-microbial interactions leading to altered concentrations of vitamin B12 [22, 48]. Another hypothesis is that genetic variants may disrupt the formation of fucosyltransferases which mediate the glycosylation of B12 binding proteins and their receptors, thus influencing vitamin B12 concentrations [22].

### Transcobalamin 2 (*TCN2*)

The *TCN2* gene also known as transcobalamin 2 is located on chromosome 22. This gene has the function of making a vitamin B12 binding protein called transcobalamin II (TC) found in human serum [49]. Data suggests that *TCN2* genetic variants are associated with Alzheimer’s disease and clinical manifestations of autoimmune gastritis in individuals with low vitamin B12 status [50, 51]. TC is involved with absorption and transporting vitamin B12 into the cell. Only 10–20% of vitamin B12 is attached to TC; the remainder is attached to holo-haptocorrin (transcobalamin 1) [18, 52, 53]. Five studies have reported associations between variants within the *TCN2* gene and vitamin B12 levels [12, 18, 22, 52, 54].

The most commonly reported *TCN2* polymorphism in Caucasian populations is the SNP rs1801198, where the C to G substitution at nucleotide 776 (TCN2 776C>G) results in an amino acid exchange of proline to arginine at codon 259 (P259R). In a candidate gene association study of 613 Irish men, a significant association was observed between the SNP rs1801198 and serum vitamin B12 levels (*P* = 0.01). Individuals with the homozygous wildtype ‘CC’ genotype had lower vitamin B12 levels (mean 243.5 pmol/l) compared to those with ‘GG’ genotype (mean 279.7 pmol/l) [54]. In contrast, it was observed that holo-transcobalamin (Holo-TC) concentrations were significantly associated with the SNP rs1801198, in a population of 122 individuals from Portugal, where the G allele carriers (median 54.2 pmol/l) had lower Holo-TC levels compared to the C variant (*P* < 0.05; median 66.3 pmol/l) [52]. Four other studies reported no significant associations between the SNP rs1801198 and vitamin B12 concentrations in Caucasian populations (*P* > 0.05) [55–58]. It was found that the minor allele frequency (G allele) of the SNP rs1801198 ranged between 35 and 48% in Brazillian (36%) [58], Latino (35%) [56], Nordic (44%) [55, 57], Northern Irish (45%) [54] and Portuguese (48%) [52] individuals. Additional variants of the *TCN2* gene (rs757874, rs4820888, rs1131603 and rs5753231) were associated with vitamin B12 status (*P* < 0.05) in individuals of Indian, Canadian, US, African American and Scandinavian background [12, 18, 22, 55, 59].

It has been suggested that the 776GG homozygous variant encodes a protein with a lower binding affinity to vitamin B12 in comparison to the wildtype ‘C’ allele [56]. Additionally, other studies have indicated that variations in the TC protein reduce the binding of vitamin B12 to TC or prevent the TC-R from recognising the vitamin B12-TC complex [5].

## Genes that code for membrane transporters that actively facilitate membrane crossing

### Cubulin (*CUBN*)

Cubulin (*CUBN*) also known as the intestinal intrinsic factor receptor or intrinsic factor-cobalamin (IF-B12) receptor is located on chromosome 10. *CUBN* is expressed on the intestinal and kidney epithelial cells and is involved with the uptake of the intrinsic factor-vitamin B12 (vitaminB12-IF) complex [20, 60, 61]. *CUBN* polymorphisms have been associated with maternal neural tube defects risk, megaloblastic anaemia, coronary heart disease and gastric cancer in individuals with low vitamin B12 status [62–66].

Studies of the association between vitamin B12 status and the variants within *CUBN* have yielded conficting results. Hazra et al. [20] was the first to report an association between the ‘G’ allele of the rs1801222 (Ser253Phe) variant and higher vitamin B12 status (*β* = 0.05 pg/ml, *P* = 2.87 × 10^−9^) in 4763 individuals from the US population [20]. This association was confirmed in another study looking at 45,571 Icelandic and Danish individuals (*β* = 0.10–0.17 pmol/l; *P* = 3.3 × 10^−75^) [12]. In contrast, a study in 3114 Canadian individuals (85% Caucasian and 15% non-Caucasian) showed that the ‘G’ allele of the rs1801222 variant was associated with a higher risk of vitamin B12 deficiency (OR 1.61 pmol/l, 95% CI 1.24–2.09, *P* = 3.0 × 10^−4^) [18]. Genotypic frequency of the risk conferring minor allele ‘A’ was compared between three different studies (Canadian, Nordic and individuals of European ancestry living in the USA). It was found that Canadian individuals carried the lowest frequency of the risk allele ‘A’, at 10% [18]. On the other hand, Hazra et al. [20] and Grarup et al. [12] observed that the minor allele frequency ‘A’ was 28.0 and 40.7% in Caucasian individuals residing in the USA and Nordic populations, respectively. Interestingly, several other genetic variants within *CUBN* (rs4748353, rs11254363 and rs12243895) were found to be either positively or negatively associated with vitamin B12 levels in residents from China, [19] Canada [18], USA and Italy [21].

To date several hypotheses have attempted to explain how *CUBN* variants are involved with lower vitamin B12 concentrations. One hypothesis is that *CUBN* is co-expressed with the protein amnionless (*AMN*, chromosome 14) forming the cubam complex [67]. Cubilin has additionally been suggested to function together with megalin (*LRP2*, chromosome 2) [68], thus any polymorphisms in either *AMN* or *LRP2* genes can affect B12 absorption leading to B12 malabsorption and deficiency. Another hypothesis is that polymorphisms affecting CUBN decrease the transport and the absorption of vitamin B12 in the ileum [20]. Functional studies on rs11254363, rs1801222, rs12243895 and rs4748353 are required to explain how these variants affect the regulation of the *CUBN* gene.

### ATP-binding cassette subfamily D member 4 (ABCD4)

The ATP-binding cassette subfamily D member 4 (*ABCD4*) gene is located on chromosome 14. This gene codes for the ABCD4 protein, which is a membrane transporter involved in transporting vitamin B12 out of lysosomes [69]. It has been shown that polymorphisms of the *ABCD4* gene affect the functioning of the ABCD4 protein and the intracellular processing of vitamin B12 [70].

There has been only one study to date investigating the association between vitamin B12 status and *ABCD4* variants. Grarup et al. [12] examined 45,571 Nordic adults and 25,960 Icelandic adults in a GWA study [12], where the ‘T’ allele of the rs3742801 and ‘C’ allele of the rs4619337 variants were associated with higher vitamin B12 levels (*β* = 0.045–0.093 pmol/l, *P* = 5.3 × 10^−8^; *β* = 0.05, *P* = 3.4 × 10^−8^, respectively), suggesting an impact of this gene on vitamin B12 status.

Previous research has shown that the protein LMBD1 (which is responsible for the lysosomal export of vitamin B12) interacts with the ABCD4 protein. The mechanisms of interaction between LMBD1 and ABCD4 remain unclear, but it is believed that polymorphisms in human *LMBRD1* gene and *ABCD4* can prevent translocation of the vitamin B12 from the lysosome to the cytoplasm [70, 71].

### *CD320* molecule (*CD320*)

The *CD320* gene also known as the ‘*CD320* molecule’ gene is located on chromosome 19. This gene codes for the transcobalamin receptor (*TCblR*), which binds and engulfs Holo-TC by endocytosis [72]. At present, two SNPs, rs2336573 and rs8109720, have shown association with vitamin B12 levels [12, 18, 59].

The most commonly studied variant of the *CD320* gene is the rs2336573 variant, a missense polymorphism, that results in an amino acid change from glycine to arginine, at the codon position 220. Zinck et al. found that the ‘C’ allele of the rs2336573 variant was associated with a lower risk (OR 0.62, 95% CI 0.45–0.86, *P* = 0.003) of vitamin B12 below adequate (< 220 pmol/l) among 3114 Canadian adults [18]. In contrast, an earlier study looking at a population of 45,571 adults from Iceland and Denmark found that the ‘T’ allele was associated with higher B12 levels (*β* = 0.22–0.32 pmol/l; *P* = 8.4 × 10^−59^) [12]. A previous study has shown that this polymorphism is associated with the maternal risk of developing neural tube defects [62]. Cell culture models have shown that SNPs in the CD320 receptor can lead to a reduction in vitamin B12 uptake [72].

## Involved in the catalysis of enzymatic reactions in the one carbon cycle

### Methylenetetrahydrofolate reductase (*MTHFR*)

The methylenetetrahydrofolate reductase (*MTHFR*) gene is located on chromosome 1 [73] and codes for a critical enzyme involved in homocysteine remethylation. MTHFR catalyzes the reduction of 5,10-methylenetetrahydrofolate to 5-methyltetrahydrofolate in an irreversible reaction [74]. The two most well-known *MTHFR* gene polymorphisms are the C677T (rs1801133) and A1298C (rs1801131) variants. Both variants have been associated with reduced enzyme activity and an altered distribution of intracellular folate [75, 76].

The majority of candidate gene association studies have shown no association (*P* > 0.05) with *MTHFR* gene polymorphisms (rs1801131 and rs1801133) and vitamin B12 concentrations in Brazillian [77, 78], North European [28], French [79], Norweigian [80] and Spanish [81] populations. However, Thuesen, et al. reported that ‘T’ allele carriers of the C677T genotype variant were associated with an increased prevalence of low-serum vitamin B12 (OR 1·78; 95% CI 1·25, 2·54; *P* = 0·003) in a population of 6784 Danish adults [57]. There are no explanations to date, which have linked the biological mechanism of TT homozygosity and B12 deficiency. It could be postulated that the C677T polymorphism is associated with a decrease in intestinal absorption of vitamin B12 [82].

### Methioninesynthase reductase (*MTRR*)

The *MTRR* gene, also known as the ‘methionine synthase reductase’ gene is located on chromosome 5. This gene is responsible for maintaining adequate levels of activated vitamin B12 (methylcob(III)alamin), which maintains the enzyme methionine synthase in its active state [83]. Currently, four SNPs, rs162036, rs162048, rs1532268 and rs3776455, have shown associations with vitamin B12 levels in healthy individuals [13].

The first SNP *MTRR* rs162036 (Lys350Arg) is a missense polymorphism [84], which was found to be associated with vitamin B12 levels (*P* = 4.00 × 10^−2^) in 262 women of North European descent (no effect size available) [13]. The same authors, also identified a significant association (*P* < 0.05) between the SNPs rs162048, rs1532268 and rs3776455 with vitamin B12 levels. This study provides the first evidence that *MTRR* polymorphisms (rs162036, rs162048, rs1532268 and rs3776455) significantly influence the circulating vitamin B12 concentrations.

## Involved in cell cycle regulation

### Membrane-spanning 4-domains A3 (*MS4A3)*

The membrane-spanning 4-domains A3 (*MS4A3*) gene is located on chromosome 11, and codes for the MS4A3 protein (also called HTm4). It has been suggested from limited studies that the MS4A3 protein may play a role in cell cycle regulation of hematopoietic cell development by inhibiting the G(1)-S cell cycle transition [85]. The only studied variant within this gene in relation to vitamin B12 concentrations is rs2298585, which was investigated in 3495 men, all of Chinese origin. In this study [19], the ‘T’ allele of the rs2298585 variant was associated with higher serum vitamin B12 concentrations (*β =* 71.80 pg/ml, *P* = 2.64 × 10^−15^) [19]. Another study investigated this SNP in 37,283 Icelandic individuals but found no statistical significance (*β* = 0.214 pmol/l, *P* = 0.075) [12].

It has been suggested that polymorphisms of the *MS4A3* gene may affect the cell-cycle regulation in the GI tract, thus affecting the renewal of intestinal and gastric epithelial cells leading to vitamin B12 malabsorption [86]. However, data from animal studies have demonstrated that MS4A3 is restricted to differentiating cells in the central nervous system and hematopoietic cells [87].

## Mitochondrial protein

### Methylmalonic aciduria (cobalamin deficiency) cb1A type (*MMAA*)

The *MMAA* gene also known as the ‘methylmalonic aciduria (cobalamin deficiency) cb1A type’, is located on chromosome 4q31.1-2 [88]. *MMAA* encodes a protein (MMAA) that may be involved in the translocation of vitamin B12 into the mitochondria [89]. In addition, MMAA could play an important role in the protection and reactivation of methylmalonyl-coA mutase (MCM) in vitro [90]. Three studies have reported associations between variants within the *MMAA* gene and vitamin B12 concentrations [12, 13, 22].

Andrew et al. was first to report that the SNP rs4835012 was significantly associated with vitamin B12 concentrations (*P* = 3.00 × 10^−2^) in 262 Caucasian women of North European descent (no effect size available) [13]. More recently in a GWA study looking at 534 Indian children, the ‘C’ allele of the SNP rs2270655 was significantly associated with lower vitamin B12 concentrations (*β* = − 0.20 pmol/l, *P* = 2.00 × 10^−2^) [22]. This association was confirmed in another study looking at 45,576 Danish and Icelandic adults (*β* = − 0.07 to − 0.30, *P* = 2.20 × 10^−13^) [12]. While these SNPs might be involved with determination of vitamin B12 concentrations, their precise biochemical role is unknown.

### Methylmalonyl-CoA mutase (MUT)

The *MUT* gene also known as the methylmalonyl-CoA mutase is located on chromosome 6. The *MUT* gene provides instructions for the formation of methylmalonyl-CoA mutase (MUT), which is a mitochondrial enzyme. MUT acts as a catalyst which isomerizes methylmalonyl-CoA to succinyl-CoA [91]. MUT requires 5-prime-deoxyadenosylcobalamin (AdoCbl), which is a form of B12 that works with MUT to form succinyl-CoA. Succinyl-CoA participates in the TCA cycle (tricarboxylic cycle) to yield energy [92]. The *MUT* gene is involved in homocysteine metabolism, and it is dependent on vitamin B12 for its function [93]. Four studies have reported associations between variants within the *MUT* gene (chr6:49,508,102, rs1141321, rs9473555, rs6458690 and rs9381784) and vitamin B12 status [12, 13, 19, 20].

In a meta-analysis of data from 4763 Caucasian individuals from the USA, participants homozygous for the rs9473558 (now merged into rs1141321) ‘T’ allele (*β* = − 0.04 pg/ml, *P* = 4.05 × 10^−8^) and *MUT* rs9473555 ‘C’ allele (*β* = − 0.04 pg/ml, *P* = 4.91 × 10^−8^) were inversely associated with plasma vitamin B12 levels [20]. These findings were confirmed in other studies involving Icelandic (*β =* − 0.061 pmol/l; *β* = − 0.062 pmol/l, repectively) [12] and Chinese populations (*β* = − 30.34 pg/ml; *β* = − 31.0 pg/ml, respectively) [19].

### Citrate lyase beta like (*CLYBL*)

The citrate lyase beta like (*CLYBL*) gene is located at chromosome 13 and codes for a human mitochondrial protein. The functions of CLYBL include metal ion binding, carbon-carbon lyase activity and citrate (pro-3s)-lyase activity [19]. Approximately, 5% of humans have a stop codon polymorphism in *CLYBL* which is associated with low levels of plasma vitamin B12, but the mechanistic link of this to vitamin B12 is currently unknown [94].

The association between the *CLYBL* variant rs41281112 and vitamin B12 levels has been studied in two different populations. Lin et al. [19] found that the ‘T’ allele was associated with lower serum vitamin B12 levels among 3495 men of Chinese Han and Chinese descent (*β =* − 83.60 pg/ml, *P* = 9.23 × 10^−10^) [19]. Similarly, Grarup et al. [12] found that the ‘T’ allele of the SNP rs41281112 variant was associated with lower serum vitamin B12 levels (*β = −* 0.29 to *−* 0.17 pmol/l, *P* = 8.9 × 10^−35^) in 45,571 adults, all of Icelandic and Danish origin [12].

At present, molecular functioning studies have elucidated that the polymorphism rs41281112 (G<A) changes the amino acid from Arginine to a stop codon resulting in a loss of protein expression [94]. As a result, Lin et al. [19] proposed that the rs41281112 variant interferes with the binding of *CLYBL* protein to metal ions, potentially leading to a lower uptake of vitamin B12 [19].

### Other genes

Our review also identified that SNPs in actin like 9 (*ACTL9*, rs2340550) [19], serum paraoxonase/arylesterase 1 (*PON1*, *rs391757*) [18], cystathionine beta synthase (*CBS*, *rs2124459*) [18], carbamoyl-phosphate synthase 1 (*CPS1*, rs1047891) [12] and DNA methyltransferase gene/ tRNA aspartic acid methyltransferase 1 (*DNMT2/TRDMT1*, rs56077122 [12] and rs2295809 [18]) genes were associated with vitamin B12 status in Canadian, Chinese, Danish and Icelandic populations. The SNPs in the intergenic regions [rs583228, rs10515552, rs12377462 [19], rs117456053, rs62515066 and Chr6:88,792,234 [12] were found to be associated with vitamin B12 status, however, plausible underlying biological mechanism as to why these SNPs were associated with vitamin B12 concentrations have not been identified.

### Ethnic-specific genetic differences in B12 deficiency

In the past, vitamin B12 deficiency within populations in the Indian subcontinent, Mexico, Central and South America and certain regions of Africa was solely attributed to dietary habits/low consumption of meat [95]. We now know that genetic factors also influence vitamin status in individuals [96]. Indian populations have a high prevalence of vitamin B12 deficiency, typically attributed to the high number of vegetarians present in the population. However, non-vegetarians in India have been observed to have lower vitamin B12 concentrations compared to Caucasian populations [27, 97]. In addition, a recent systematic review showed that B12 deficiency is common during pregnancy in other populations where vegetarianism is rare [98]. Poor dietary intake, low bioavailable B12 in meat products (i.e. food processing and reheating of food) and a possible underlying genetic predisposition to vitamin B12 status could be the reasons for such observation in non-vegetarian populations [99, 100].

Although several studies have explored the association of SNPs with vitamin B12 status, only a limited number of genetic loci have been reported to support the presence of ethnic differences in vitamin B12 status in non-European populations [19, 22]. We can assume four genetic mechanisms which possibly account for these differences: (1) difference in effect allele frequencies, (2) genetic heterogeneity across different ethnic groups, (3) variance in LD structure and (4) gene-gene and gene-environment interactions [101]. A key example of ethnic specificity has been demonstrated in the *FUT2* gene, whereby different mutations leading to nonsecretor status have been identified (the secretor status of *FUT2* gene is associated with a low vitamin B12 status) [102]. The 428G→A polymorphism (rs601338) is the characteristic for the nonsecretor allele in Europeans and appears in about 20% of the Caucasian population [103]. In South-East and East-Asians populations, the SNP rs601338 is rare and the more common *FUT2* missense mutation rs1047781 is associated with nonsecretor status [104].

Genetic variants associated with circulating vitamin B12 have been studied in the following populations: African American (*n* = 1) [59], Brazilian (*n* = 4) [58, 77, 78, 105], Canadian (*n* = 1) [18], Caucasian (*n* = 4) [20, 28, 29, 59], Chinese (*n* = 1) [19], Danish (*n* = 2) [12, 57], European ancestry (*n* = 1) [13], French (*n* = 1) [79], Icelandic (*n* = 1) [12], Indian (*n* = 2) [22, 27], Italian ancestry and residents of the USA (*n* = 1) [21], Latino (*n* = 2) [56, 81], Northern Irish (*n* = 1) [54], Norwegian (*n* = 2) [55, 80] and Portuguese (*n* = 1) [52]. To date, the majority of genetic association studies of vitamin B12 status have been performed in Caucasian populations, and a few have reported associations in high-risk populations such as Mexico and India [27, 106]. More studies exploring a wider range of ethnicities with large sample sizes may help to identify novel SNPs that may be associated with vitamin B12 status. Studying the genetic structure of chromosomal regions that are associated with variability in vitamin B12 levels in different populations can help us understand the evolutionary aspects of B12 associations and their relationship with environmental exposures. It is important that before any diet-related recommendations based on genotypes are given at the population level, associations between the SNPs and various health outcomes need to be confirmed [107].

## Conclusion

In summary, our review has identified significant associations of vitamin B12 status with 59 B12-related SNPs from 19 genes. Among these genes, five were co-factors or regulators for the transport of vitamin B12 (*FUT2*, *FUT6*, *MMACHC*, *TCN1* and *TCN2*); three were membrane transporters actively facilitating the membrane crossing of vitamin B12 (*ABCD4*, *CUBN* and *CD320*); three were involved in the catalysis of enzymatic reactions in the one-carbon cycle (*CBS, MTHFR* and *MTRR*); one was involved in cell cycle regulation (MS4A3); three were mitochondrial proteins (*CLYBL*, *MMAA* and *MUT*) and lastly four genes had an unknown function (*ACTL9*, *CPS1*, *DNMT2*/*TRDMT1* and *PON1*). Our review highlights the complex nature of the B12 genetics where several genes/SNPs from various parts of B12 metabolic pathway contribute to the susceptibility to vitamin B12 deficiency. Identification of gene variants involved in this metabolic pathway using large-scale genetic association studies in diverse ethnic populations would contribute to our understanding of the pathophysiology of B12 deficiency and help in discovering biomarkers of vitamin B12-related chronic diseases.
